# Effects of Silica Modification (Mg, Al, Ca, Ti, and Zr) on Supported Cobalt Catalysts for H_2_-Dependent CO_2_ Reduction to Metabolic Intermediates

**DOI:** 10.1021/jacs.2c08845

**Published:** 2022-11-09

**Authors:** Kendra S. Belthle, Tuğçe Beyazay, Cristina Ochoa-Hernández, Ray Miyazaki, Lucas Foppa, William F. Martin, Harun Tüysüz

**Affiliations:** † Max-Planck-Institut für Kohlenforschung, Kaiser-Wilhelm-Platz 1, 45470 Mülheim an der Ruhr, Germany; ‡ The NOMAD Laboratory at the FHI of the Max-Planck-Gesellschaft and IRIS-Adlershof of the Humboldt-Universität zu Berlin, Faradayweg 4-6, 14195 Berlin, Germany; § Institute of Molecular Evolution, University of Düsseldorf, Universitätsstraße 1, 40225 Düsseldorf, Germany

## Abstract

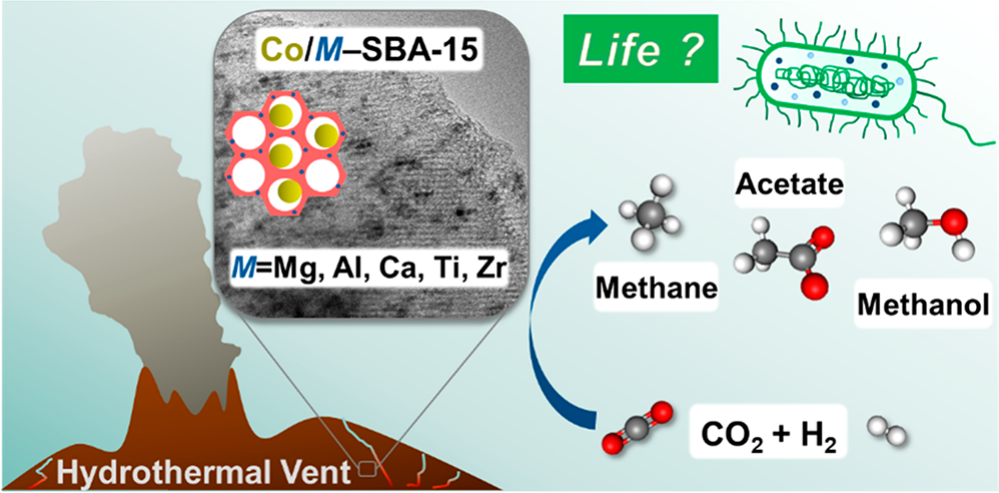

Serpentinizing hydrothermal systems generate H_2_ as a reductant and harbor catalysts conducive to geochemical CO_2_ conversion into reduced carbon compounds that form the core of microbial autotrophic metabolism. This study characterizes mineral catalysts at hydrothermal vents by investigating the interactions between catalytically active cobalt sites and silica-based support materials on H_2_-dependent CO_2_ reduction. Heteroatom incorporated (Mg, Al, Ca, Ti, and Zr), ordered mesoporous silicas are applied as model support systems for the cobalt-based catalysts. It is demonstrated that all catalysts surveyed convert CO_2_ to methane, methanol, carbon monoxide, and low-molecular-weight hydrocarbons at 180 °C and 20 bar, but with different activity and selectivity depending on the support modification. The additional analysis of the condensed product phase reveals the formation of oxygenates such as formate and acetate, which are key intermediates in the ancient acetyl-coenzyme A pathway of carbon metabolism. The Ti-incorporated catalyst yielded the highest concentrations of formate (3.6 mM) and acetate (1.2 mM) in the liquid phase. Chemisorption experiments including H_2_ temperature-programmed reduction (TPR) and CO_2_ temperature-programmed desorption (TPD) in agreement with density functional theory (DFT) calculations of the adsorption energy of CO_2_ suggest metallic cobalt as the preferential adsorption site for CO_2_ compared to hardly reducible cobalt–metal oxide interface species. The ratios of the respective cobalt species vary depending on the interaction strength with the support materials. The findings reveal robust and biologically relevant catalytic activities of silica-based transition metal minerals in H_2_-rich CO_2_ fixation, in line with the idea that autotrophic metabolism emerged at hydrothermal vents.

## 1 Introduction

Submarine hydrothermal vents have been of interest for origin of life chemistry due to their minerals’ composition as well as their thermal and chemical gradients resulting from rock–water interactions.^1−4^ Both in high temperature peridotite-hosted systems, such as Rainbow with an effluent temperature of ca. 360 °C, and in low temperature peridotite-hosted systems, such as Lost City with an effluent temperature of ca. 115 °C, H_2_ is continuously produced in the crust via water reduction by Fe^2+^ in iron magnesium silicates during a process called serpentinization.^5^ Geochemically produced H_2_ can react with CO_2_ to generate abiotic formate, CH_4_, and other hydrocarbons.^6,7^ The geochemical CO_2_ conversion catalyzed by transition metals at hydrothermal vents resembles, in terms of catalysts and products, the ancient and only exergonic pathway of autotrophic carbon metabolism, the acetyl-coenzyme A (acetyl-CoA) pathway.^8^ This biochemical CO_2_ fixation pathway of modern anaerobes is catalyzed by enzymes and cofactors that contain nickel, iron, and cobalt at their active sites,^9,10^ transition metals that naturally occur in the minerals of hydrothermal vents, sometimes as native, zerovalent metals.^11−13^ Recent studies have focused on Fe- and Ni-based catalysts for CO_2_ fixation with H_2_ under simulated hydrothermal vent conditions.^14−16^ Fe- and Ni-based catalysts yield formate, acetate, pyruvate, methanol, and CH_4_,^14^ all of which are germane to the acetyl-CoA pathway in acetogens and methanogens.^9,17^ Though less widely studied in an origins of life context, Co is also essential to the acetyl-CoA pathway as the central metal of cobamide, where it performs a biologically rare metal-to-metal methyl transfer reaction during acetyl moiety synthesis.^18^ Under hydrothermal conditions, Co catalyzes the high pressure H_2_-dependent synthesis of long-chain hydrocarbons^19^ and the low pressure reduction of *nicotinamide adenine dinucleotide* NAD^+^ to NADH.^20^

The effects of Co–support interactions are well-studied in typical industrial applications of Co-based catalysts. In the Fischer–Tropsch process for CO hydrogenation to long-chain paraffins, support effects have been shown to play a substantial role in controlling the catalytic performance.^21−23^ The Co dispersion and stabilization of the metallic cobalt nanoparticles, for example, have been shown to depend on the nature of the support material and guide the activity and selectivity of hydrocarbon production. Co–support interactions are usually weaker with silica materials as compared to alumina, resulting in lower cobalt dispersion and better cobalt reducibility.^22^ Similarly, in the CO_2_ hydrogenation reaction, the strength of Co–support interactions has been reported to control the activity and selectivity by tailoring the oxidation state of the Co-based catalysts.^24−26^ Coexisting phases of metallic Co and CoO on SiO_2_, stabilized by specific Co–support linkages in a cobalt phyllosilicate structure, have been reported to enhance methanol selectivity.^25^ The catalytic activity has typically been reported to be higher for metallic Co compared to CoO in case of silica supports, while the reversed trend has been observed for TiO_2_.^24,27^ In hydrothermal vents, the surrounding minerals and their variable compositions can also be expected to influence H_2_-dependent CO_2_ conversions by native transition metals.

Here, we have chosen ordered mesoporous SBA-15 silica as a model support material to investigate Co–support interaction effects, as the deep-sea vents mainly consist of silica-rich mixtures of serpentinized peridotite and mafic material.^5,28^ In general, the key advantages of ordered mesoporous materials as supports for heterogeneous catalysts are their high specific surface areas and uniform pore structures with tunable pore size. SBA-15 combines the controllable uniform hexagonal pore structure with thick pore walls, which result in a good mechanical strength and hydrothermal stability.^29^ Such an internal porous structure is also of importance in the context of a hydrothermal origin of metabolism. Pores in the (sub)micrometer range within minerals or at their surface might have acted as prebiotic microspaces for the concentration of the essential prebiotic molecules needed at the emergence of life and the control of the water activity to prevent hydrolysis of the biomolecules.^30,31^ Based on the cations found in the minerals at hydrothermal vents,^11,12,32^ different additional atoms like Mg, Al, Ca, Ti, and Zr are incorporated into the silica matrix during the support synthesis in this study. These high surface area materials with well-defined morphology are further loaded with cobalt nanoparticles and applied as solid catalysts for CO_2_ hydrogenation to explore the formation of intermediate metabolic products in the liquid and gas phase using a fixed-bed flow reactor.

## 2 Experimental Section

### Materials

Pluronic P123 (EO_20_:PO_70_:EO_20_, *M*_n_ = 5800, Sigma-Aldrich), tetraethyl orthosilicate (TEOS; reagent grade 98%, Sigma-Aldrich), HCl (37–38%, J.T. Baker), magnesium acetate tetrahydrate (MgAc_2_·4H_2_O; ACS reagent grade 99%, Sigma-Aldrich), aluminum isopropoxide (Al(−O^*i*^Pr)_3_; ≥98%, Sigma-Aldrich), calcium nitrate tetrahydrate (Ca(NO_3_)_2_·4H_2_O; 99%, Alfa Aesar), titanium(IV) isopropoxide (Ti(−O^*i*^Pr)_4_; 97%, Sigma-Aldrich), zirconyl chloride octahydrate (ZrOCl_2_·8H_2_O; ≥98%, Acros Organics), sodium chloride (NaCl; ACS reagent grade ≥99.0%, Sigma-Aldrich), and cobalt(II) nitrate hexahydrate (Co(NO_3_)_2_·6H_2_O; ACS reagent grade 99.5%, Sigma-Aldrich) were used as purchased.

### Support Synthesis

The mesoporous SBA-15 support was synthesized following a procedure reported in literature using Pluronic P123 as a structure-directing agent and TEOS as a silica source.^29^ In a typical synthesis, 27.8 g of the triblock copolymer were dissolved in 504 g of distilled water. A 15.5 g amount of HCl was added, and the solution was heated to 35 °C. After 1 h of stirring, 60.0 g of TEOS were added and the reaction mixture was stirred for another 24 h at 35 °C. Afterward, the solution was aged at 100 °C for 24 h under autogenous pressure. The obtained solid was filtered, washed with distilled water, and dried at 80 °C overnight. To remove the organic template, the solid was calcined at 180 °C (2 h, 5 °C min^–1^) and 550 °C (6 h, 1.5 °C min^–1^).

A series of heteroatom modified *M*–SBA-15 materials (*M* = Mg, Al, Ca, Ti, Zr) with molar ratios of Si/*M* = 10 were synthesized by direct synthesis methods reported in literature.^33−37^

In the synthesis of Mg–SBA-15, MgAc_2_·4H_2_O was used as the magnesium source.^33^ Briefly, 0.88 g of MgAc_2_·4H_2_O was added to a solution of 4.0 g of the copolymer Pluronic P123 in 30 mL of water and 120 mL of 2 M HCl and stirred for 3 h at 40 °C. An 8.5 g amount of TEOS was added dropwise, and the solution was stirred for another 24 h at 40 °C. The suspension underwent hydrothermal treatment at 100 °C for 24 h under autogenous pressure. The resulting gel was heated to 80 °C to evaporate the solvent and dried overnight. The final solid product was obtained after removal of the template during calcination in air at 550 °C for 6 h (2 °C min^–1^).

The Al–SBA-15 material was synthesized by adding 0.83 g of Al(−O^*i*^Pr)_3_ and 8.5 g of TEOS to 10 mL of HCl at pH = 1.5 and stirring for around 3 h until a homogeneous gel was obtained.^34^ The gel was added to a solution of 4.0 g of Pluronic P123 in 150 mL of HCl at pH = 1.5 at 40 °C, which was stirred for 3.5 h before. After stirring the reaction mixture for another 24 h, the gel was kept in an oven at 100 °C for 24 h for hydrothermal treatment under autogenous pressure. The solid was filtered, washed with distilled water, and dried at 60 °C overnight. Finally, the template was removed by calcination at 550 °C for 5.5 h (1.8 °C min^–1^).

For the synthesis of Ca–SBA-15, 0.96 g of the calcium source Ca(NO_3_)_2_·4H_2_O was dissolved in 30 mL of water and added to a solution of 4.0 g of Pluronic P123 in 120 mL of 2 M HCl.^35^ After stirring for 30 min at 40 °C, 8.5 g of TEOS were added dropwise and the reaction mixture was stirred for 24 h at 40 °C. Hydrothermal treatment at autogenous pressure at 100 °C for 24 h resulted in a gel, which was dried by the evaporation of the solvent at 55 °C in the rotary evaporator and at 70 °C overnight. The mesoporous solid was obtained after final calcination at 550 °C for 6 h (2 °C min^–1^).

The Ti–SBA-15 support material was synthesized using Ti(−O^*i*^Pr)_4_ as the titanium source.^36^ A 9.3 g amount of Pluronic P123 was dissolved in 229 g of water by stirring at 40 °C for 2 h. Then 4.5 g of HCl were added, and the solution was stirred for another 2 h at 40 °C. A premixed solution of 20.8 g of TEOS and 2.88 g of Ti(−O^*i*^Pr)_4_ was added dropwise, and the reaction mixture was stirred for 24 h at 40 °C. The resulting gel was aged at 100 °C for 24 h under autogenous pressure. The solid product was then filtered off, washed with water, and dried at 100 °C overnight. Finally, the solid was calcined in air at 550 °C for 6 h (1.5 °C min^–1^) to remove the organic template.

Zr–SBA-15 was synthesized from ZrOCl_2_·8H_2_O as a zirconium source.^37^ A 4.0 g amount of triblock copolymer P123 was dissolved in 160 g of water, and 2.4 g of NaCl were added. After stirring for 2 h at 35 °C, 1.31 g of ZrOCl_2_·8H_2_O was added and the solution was stirred for another 24 h at 35 °C. The reaction mixture was then aged at 100 °C for 24 h under autogenous pressure. Afterward, the solid product was filtered off, washed with water, and dried at 80 °C. The final product was obtained after removal of the polymeric template by calcination at 550 °C for 12 h (1 °C min^–1^).

### Catalyst Synthesis

The 10 wt % Co-loaded *M*–SBA-15 (*M* = Mg, Al, Ca, Ti, Zr) catalysts were synthesized by a classical wet impregnation/reduction procedure. Typically, 1.35 g of the support material was dispersed in a solution of 782 mg of Co(NO_3_)_2_·6H_2_O in 15 mL of EtOH and stirred for 2 h at room temperature. Subsequently, it was dried at 45 °C in air overnight, followed by reduction at 200 °C for 2 h and 450 °C for 8 h in 100 cm^3^ min^–1^ H_2_ flow (1 °C min^–1^). Afterward, surface passivation of the metallic cobalt nanoparticles was performed in 90 cm^3^ min^–1^ Ar/10 cm^3^ min^–1^ synthetic air flow to prevent unregulated oxidation upon exposure to air. The catalysts are in the following referred as Co/*M*–SBA-15 where *M* = Mg, Al, Ca, Ti, Zr. In addition, catalysts with 5 and 20 wt % Co-loading were synthesized on SBA-15.

### Characterization of Supports and Catalysts

Powder X-ray diffraction (XRD) patterns were collected with a STOE theta/theta diffractometer in Bragg–Brentano geometry and a Cu Kα_1,2_ radiation source at room temperature. Bulk scanning electron microscopy coupled with energy dispersive X-ray spectroscopy (SEM–EDX) was performed with a Hitachi S-3500 N equipped with a Si(Li) Pentafet plus detector from Oxford Instruments operated at 30 kV. Dark-field high resolution transmission electron microscopy (HR-STEM) micrographs and SEM-EDX spatial elemental distribution were collected on a Hitachi S-5500 ultrahigh resolution cold field emission scanning electron microscope at a maximum acceleration voltage of 30 kV. The cross-sectional cuttings were obtained by embedding the materials in Spurr-resin and cutting in an ultramicrotrome (Reichert Ultracut) equipped with a diamond knife. X-ray photoelectron spectroscopy (XPS) was performed using a SPECS spectrometer with a hemispherical analyzer (PHOIBOS 150). The monochromatized Al Kα X-ray source (*E* = 1486.6 eV) was operated at 15 kV and 200 W. As lens mode the medium area mode was used. The base pressure in the analysis chamber during the experiment was 5 × 10^–10^ mbar. The C 1s peak for contaminant carbon was used as a reference at 284.5 eV for correction of the binding energy for surface charging. Nitrogen physisorption measurements were carried out on a 3Flex Micrometrics at −196 °C. The samples were degassed at 150 °C for 10 h under vacuum prior to the measurement. Specific surface areas (*S*_BET_) were calculated with the Brunauer–Emmet–Teller (BET) method within the relative pressure range *p*/*p*_0_ = 0.05–0.2. The pore volume (*V*_p_) was determined from the adsorption branch of the isotherms at *p*/*p*_0_ = 0.95. The pore size distribution was determined from the adsorption branch of the isotherms by the Barrett–Joyner–Halenda (BJH) method. Transmission electron microscopy (TEM) micrographs of the catalysts and high-resolution images were taken using an electron microscope HF-2000 from Hitachi operated at 200 kV acceleration voltage with a cold field emission gun (FEG). Chemisorption experiments were performed in a Micromeritics AutoChem II 2910. For hydrogen temperature-programmed reduction (H_2_-TPR) experiments, the calcined Co-based catalysts (calcination at 400 °C for 4 h in 200 cm^3^ h^–1^ g_cat_^–1^ air flow, 1 °C min^–1^) were pelletized and sieved to 200–400 μm grain size. About 80 mg of the sieve fraction were pretreated at 200 °C in 50 cm^3^ min^–1^ He flow for 60 min (10 °C min^–1^). After cooling down to 45 °C (5 °C min^–1^), the gas flow was switched to 10 vol % H_2_ in Ar (50 cm^3^ min^–1^) and the temperature was increased to 800 °C at a heating rate of 10 °C min^–1^. The H_2_ consumption rate was monitored by a thermal conductivity detector (TCD). The water generated during the reduction was retained by an acetone/dry ice trap. Temperature-programmed desorption of carbon dioxide (CO_2_-TPD) was performed with about 80 mg of the catalyst sieve fraction (200–400 μm grain size). The catalysts were reduced in situ in 10% H_2_/Ar flow (50 cm^3^ min^–1^) at 450 °C for 2 h (5 °C min^–1^). The gas flow was switched to He (50 cm^3^ min^–1^) to remove any physisorbed H_2_, and the sample was cooled down to 50 °C (10 °C min^–1^). CO_2_ was chemisorbed by exposing the sample to 10 vol % CO_2_/He flow (50 cm^3^ min^–1^) for 30 min. Afterward, the sample was purged with He (25 cm^3^ min^–1^) for 65 min to remove weakly adsorbed CO_2_ prior to heating to 450 °C at 10 °C min^–1^. The CO_2_ desorption profile was tracked at *m*/*z* = 44 by an InProcess Instruments quadrupole mass spectrometer GAM2000 connected downstream of the chemisorption setup. The total amount of acid sites of the support materials was analyzed by temperature-programmed desorption of ammonia (NH_3_-TPD). About 80 mg of sample sieved to 200–400 μm grain size were heated to 550 °C for 120 min in 50 cm^3^ min^–1^ Ar flow (10 °C min^–1^) to remove physisorbed water from the surface. After cooling down and flushing with He for 20 min (50 cm^3^ min^–1^), NH_3_ was adsorbed at 150 °C by purging 10 vol % NH_3_/He for 30 min. Once the weakly adsorbed NH_3_ was removed by flushing He for 60 min at 50 cm^3^ min^–1^ and the sample was cooled down to 100 °C, the NH_3_ desorption rate was monitored by a TCD while heating the sample to 550 °C (10 °C min^–1^). The type and strength of the acid sites were assessed by pyridine adsorption/desorption monitored by transmission Fourier-transform infrared spectroscopy (FTIR). Self-supporting wafers (∼25 mg) were outgassed at 450 °C for 4 h under vacuum in a custom-made cell with CaF_2_ windows. Pyridine adsorption was performed by introducing 3 mbar of the probe molecule into the system at 150 °C and isolating the cell for 20 min. Thereafter, a stepwise thermo-desorption process was carried out in the range 150–450 °C and under vacuum (equilibration time of 20 min at each temperature). FTIR spectra were recorded at room temperature with a resolution of 4 cm^–1^ and 128 scans per spectrum in the 4000–1200 cm^–1^ range using a Nicolet iS50 spectrometer equipped with an MCT/B detector. A spectral subtraction method and normalization to 10 mg cm^–2^ wafer density were applied to the measurements.

### Catalytic CO_2_ Hydrogenation

The catalytic performance testing was performed using an in-house built high-pressure setup (for details see Figure EM1 in the Supporting Information (SI) in S1. Experimental methods). An 850 mg amount of catalyst sieved to 200–400 μm grain size was loaded into a stainless-steel reactor. The catalyst was first reduced at 450 °C for 8 h in 200 cm^3^ STP min^–1^ H_2_ flow at ambient pressure to remove the surface passivation layer on the Co-based nanoparticles. After the reactor was cooled down to 160 °C, the gas flow was switched to the reactant gas mixture (H_2_/CO_2_/Ar = 60:30:10, Ar as internal GC standard; 56.7 cm^3^ STP min^–1^; 4000 cm^3^ h^–1^ g_cat_^–1^). Pressure was built up to 20 MPa controlled by a membrane dome regulator, and the reactor temperature was increased to 180 °C with a 1 °C min^–1^ heating ramp. The outlet gas was analyzed by an online GC Agilent 7890B equipped with two TCDs and one flame ionization detector (FID) (SI Figure EM2). The data for the conversion and selectivity after 36 h time-on-stream are presented to ensure a steady reaction state. Higher boiling oxygenate products were collected in a high-pressure trap downstream the reactor at 50 °C for 72 h time-on-stream and analyzed by high-performance liquid chromatography (HPLC) and nuclear magnetic resonance (NMR). The concentration of the products was determined by HPLC with a Shimadzu LC-2030 equipped with a Metacarb column (300 mm × 7.5 mm) coupled with a refractive index (RI) detector operated at 50 °C. As the mobile phase 0.1% trifluoroacetic acid (TFA) at a flow rate of 0.8 cm^3^ min^–1^ was used. For additional qualitative analysis of the liquid phase products, ^1^H NMR spectra were acquired on a Bruker Avance III spectrometer operated at 600 MHz and equipped with a cryogenically cooled TCI probehead. Water suppression at 4.68 ppm was enabled by “excitation sculpting”^38^ in combination with a “perfect echo”^39^ using the Bruker standard pulse-program “zgesgppe”.

### Computational Details

Spin-polarized density functional theory (DFT) calculations were performed by using the FHI-aims code (version: 210226).^40^ The RPBE functional^41^ was used to approximate the exchange-correlation interaction. The default “light” basis set was adopted for geometry optimizations, and single-point energy calculations of the optimized structures were performed with the default “tight” basis set.^40^ The lattice constants of bulk HCP cobalt are well represented by this setting (deviation from the experimental values:^42^*a* = −0.01 Å, *c* = −0.03 Å). A Γ-centered grid of 3 × 3 × 1 k-points was used for the slab models while the isolated CO_2_ molecule was calculated by a single k-point with a large unit cell where each CO_2_ is separated by >20 Å. The atomic-scaled zeroth-order regular approximation (Atomic ZORA)^40^ was adopted to incorporate relativistic effects. The most stable structure of the Co_20_ cluster in the gas phase reported in the theoretical work by Farkaš et al.^43^ was adopted as the initial structure for geometry optimizations in each model. A periodic surface slab model of the amorphous silica reported by Comas-Vives^44^ was used with 7.2 OH per nm^2^ silanol coverage, which is the most stable surface structure under the experimental calcination conditions. The Co_20_ cluster on the silica slab is found to assume a geometry that enables the formation of Co–O–SiO_*x*_ bonds (more details are shown in the SI in S2. Computational methods). The Co_20_/SiO_2_ model had a 21.4 Å × 21.4 Å × 34.2 Å unit cell including a 20 Å vacuum region perpendicular to the silica surface. For the initial spin state of the Co_20_ cluster we used a ferro-magnetic ordering. All atoms and spins in the model were relaxed during the geometry optimizations. The charge state of the cobalt atoms was estimated by evaluating the Hirshfeld charges^45^ where the total electron density is partitioned by each atomic electron density as obtained from the isolated elements. The adsorption energy of CO_2_ (*E*_ads_) was calculated by using the following equation:
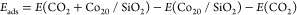
where *E*(CO_2_ + Co_20_/SiO_2_) is the potential energy of the adsorption structure of CO_2_ on the Co_20_/SiO_2_ model, and *E*(Co_20_/SiO_2_) and *E*(CO_2_) are the potential energies of the isolated Co_20_/SiO_2_ model and CO_2_, respectively.

## 3 Results

### Catalyst Synthesis and Characterization

The effect of different cobalt–support interactions on CO_2_ hydrogenation was systematically investigated by comparing Co-based nanoparticles supported on pristine SBA-15 silica and modified SBA-15 with different heteroelements (Mg, Al, Ca, Ti, Zr). The series of cations incorporated into the silica structure has been selected on the basis of the constituents of minerals occurring at hydrothermal vents.^11,12,32^ The well-defined model metal oxide support materials were synthesized via the soft-templating method with direct incorporation of the additional cations, followed by in-depth characterization. Two-dimensionally well-ordered support structures were obtained for the SBA-15 silica and heteroelement incorporated materials. The typical *p*6*mm* symmetry was evidenced from the low-angle X-ray diffraction (XRD) patterns with the characteristic reflections indexed to the (100), (110), and (200) plane^29^ (see SI Figure S1a). In the case of Ti*–*SBA-15, a slight disorder in the hexagonal symmetry was observed, which was associated with the formation of crystalline TiO_2_ particles within the support matrix as evidenced from the distinct reflections of the anatase phase of TiO_2_ in the wide-angle XRD pattern (Figure S1b). The formation of aggregates of TiO_2_ in addition to framework Si*–*O*–*Ti has been reported frequently for the direct synthesis of Ti–SBA-15 at comparable Si/Ti ratios due to the faster hydrolysis rate of the titanium alkoxide compared to the silicon precursor.^36,46,47^ All other materials possess only amorphous phases, as no characteristic reflections of the respective metal oxides have been observed in the wide-angle range of the XRD patterns.

The homogeneous distribution of all additional sites—except of Ti—was further confirmed by the spatial element distributions in the elemental mapping images from scanning electron microscopy coupled with energy dispersive X-ray spectroscopy (SEM–EDX) in Figure S2a–e. The observation of additional TiO_2_ particles in Ti*–*SBA-15 is in line with the XRD data. The determined Si/*M* molar ratios of 9–14 (*M* denotes the heteroelement) agree with the intended value of 10 (Table S1). However, the much higher ratios of Si/Mg = 31, Si/Al = 31, and Si/Ca = 22 calculated from X-ray photoelectron spectroscopy (XPS) indicate lower concentrations of the respective cations at the surface of the support materials compared to the bulk structure (Table S1 and Figure S3a–j). The similar physicochemical properties of the support materials stress their suitability as model support materials to study Co–support interactions without additional effects by diverse porosity. All silica materials possess type-IV N_2_ physisorption isotherms with a H1 hysteresis loop,^48^ high specific surface areas (464–905 m^2^ g^–1^), pore volumes (0.73–1.21 cm^3^ g^–1^), and comparable pore size distributions in the mesoporous range of 7.7–12.5 nm (Table S1 and Figure S4).

By using these ordered mesoporous materials as supports, similar Co particle sizes were intended to avoid interfering particle size effects on the catalytic performance. In addition to 10 wt % Co nanoparticles on different support materials, catalysts with a 5 and 20 wt % Co loading on regular SBA-15 silica were synthesized to explore the effect of the metal loading on CO_2_ reduction. The Co loadings were determined by SEM–EDX analysis as 6.0 and 24.0 wt % for the last-mentioned catalysts and 11.0–12.7 wt % for the different support materials (Tables S2, S3). Despite the small deviations from the intended Co loading, the catalysts are referred to as 5, 10, and 20 wt % Co/*M*–SBA-15 in the following for clarity.

The cobalt nitrate precursor was reduced in a H_2_ atmosphere to obtain the zerovalent metallic phase. After the reduction, all catalysts contained the metallic fcc Co phase and in addition the CoO phase as assessed by the XRD patterns (Figure S5a, b). The cobalt oxide phase can be formed upon exposure to ambient air during the passivation step, and it can also result from incomplete reduction of the Co precursor. While similar reflection patterns for the Co-based nanoparticles on the different support materials were obtained, sharper reflections of both Co and CoO phases indicated the formation of larger crystallites at higher Co loadings with increasing size of the Co particles. For all catalysts, the pore structure was preserved when loaded with Co as evidenced from the type-IV N_2_ physisorption isotherms, but the mode of the pore diameter was slightly decreased (Figure S6a, b and Supplementary Tables S2, S3).

**Figure 1:**
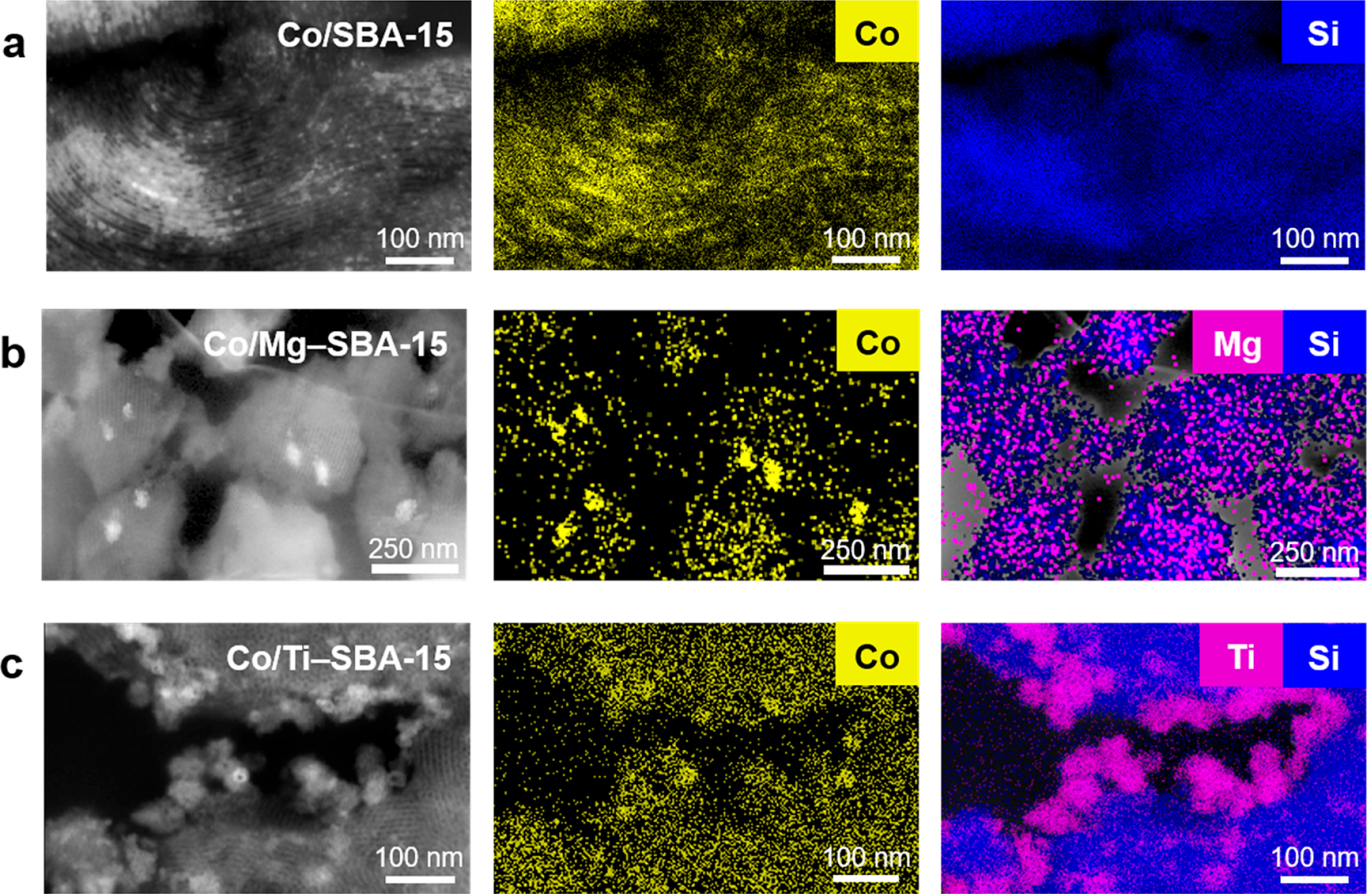
Dark-field high resolution scanning transmission electron microscopy (HR-STEM) micrographs and energy dispersive X-ray spectroscopy (EDX) elemental mappings of selected catalysts 10 wt % (a) Co/SBA-15, (b) Co/Mg–SBA-15, and (c) Co/Ti–SBA-15.

The morphology of the materials and location of the Co-based particles were further investigated by SEM-EDX elemental mappings. Figure 1a–c show micrographs and elemental mappings of selected 10 wt % Co/*M*–SBA-15 catalysts. The imaging and EDX analyses indicate a homogeneous distribution of the Co nanoparticles within the SBA-15 silica structure. The mean particle diameter determined from the TEM micrographs agreed well with the pore diameter for 10 wt % Co/SBA-15 (8.8 nm), while a smaller particle size was obtained at the lower loading of 5 wt % Co/SBA-15 (5.7 nm) (Figure S7). 20 wt % Co/SBA-15 showed a bimodal particle size distribution due to the formation of aggregates (22–38 nm) consisting of smaller cobalt crystals at the surface of the support material in addition to the particles inside the pores. For the 10 wt % Co/*M*–SBA-15 catalysts with the modified supports mean Co particle diameters in the range of 5.8–8.8 nm were determined from the TEM micrographs (Figure S8a–j), which indicates the formation of the particles inside the cylindrical pores with a comparable size range. However, agglomeration of some of the Co particles was observed with the modified supports.

### CO_2_ Hydrogenation toward Metabolic Intermediates

In previous work, we demonstrated the ability of nanostructured Fe-based native metal alloys to convert CO_2_ to formate, acetate, methanol, and pyruvate in a batch reactor at 60–100 °C and a pressure of 2.5 MPa.^14^ In this study, the reaction with the supported Co-based catalysts was performed in a continuous flow setup with a fixed-bed reactor at 180 °C and 2.0 MPa, which is also in the temperature and pressure range expected at hydrothermal vents. The present reaction design allows us to investigate mineral catalyzed CO_2_ hydrogenation reactions under conditions of reduced water activity.^30^

The gas phase products were monitored over time by online gas chromatography (GC). In addition, higher boiling point products were collected in a cold trap at 50 °C and analyzed offline by high-performance liquid chromatography (HPLC) and nuclear magnetic resonance (NMR). These oxygenates are of particular interest for the comparison with CO_2_ fixation products from microbial metabolism. Based on the temperature range reported for the effluents of different hydrothermal systems,^5,49^ reaction temperatures between 160–340 °C have been investigated with the 10 wt % Co/SBA-15 catalyst. Within this temperature range, the selectivity toward oxygenates decreased with increasing reaction temperature and CH_4_ was formed as the main product with a selectivity >90% at *T* ≥ 200 °C (Figure S9a, Table S4). At low CO_2_ conversions (*T* < 180 °C), the volume of the liquid phase products collected in the cold trap was not sufficient for proper product analysis (<100 μL). Thus, the reaction temperature of 180 °C appeared most suitable to study the effect of different supports on the catalytic performance of Co catalysts in CO_2_ hydrogenation to metabolic intermediates.

**Figure 2:**
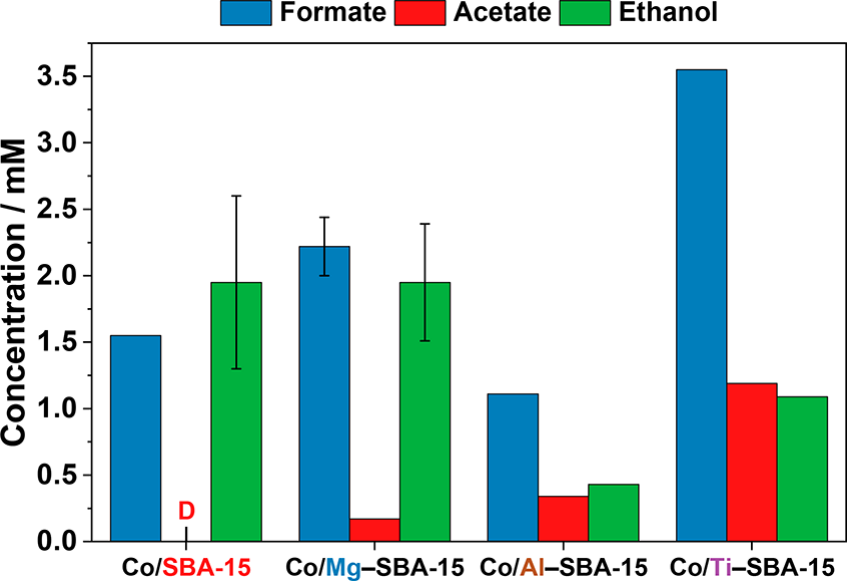
HPLC results for concentration of oxygenate products from CO_2_ hydrogenation with 10 wt % Co/*M*–SBA-15 catalysts (*M* = Mg, Al, Ti) collected after 72 h time-on-stream. D: detected, concentration <0.2 mM. Reaction conditions: *T* = 180 °C, *p* = 2.0 MPa, H_2_/CO_2_/Ar = 6:3:1, 4000 cm^3^ h^–1^ g_cat_^–1^. Exemplary error bars are shown based on the reproduction of the reaction with different catalyst batches and the measurement of the liquid phase sample at different times-on-stream. The deviation for acetate was below the accuracy of the method (±0.1 mM), as well as the deviation for formate in case of Co/SBA-15.

To provide insights into the formation of oxygenate products, we first discuss the results of the analysis of the condensed product phase. In the comparison of the Co/SBA-15 catalysts with varying Co loadings, the 5 wt % catalyst yielded too little product for liquid phase analysis, while the 10 and 20 wt % Co catalysts formed formate, acetate, and ethanol according to HPLC and ^1^H NMR analyses results (Figure S10, Table S5). Additionally, methanol was detected by HPLC, but it was more quantitatively analyzed in the gas phase products and will be discussed in detail later. The formation of acetate from CO_2_ is of particular interest, as the formation of an activated acetic acid via the acetyl-CoA pathway has appeared to be the key step for the initial carbon metabolism of microorganisms.^9^ Formate, however, is detected as the main organic product in the effluent of hydrothermal vents,^50^ and the formation of a formyl group from CO_2_ reduction is also the initial carboxylation step of the ancient metabolic acetyl-CoA pathway.^10,51^ Thus, our study confirms the ability of silica-supported Co-based catalysts to convert CO_2_ similarly to the biological route catalyzed by enzymes with Co active centers. The concentration of formate decreased from 1.6 mM to 0.4 mM with increasing Co loading from 10 wt % to 20 wt %, indicating a shift in the selectivity toward enhanced formation of water as a byproduct compared to formic acid. The Co loading of 10 wt % was therefore chosen as the optimal composition for the comparison of the different support materials. In the case of the Ca- and Zr-containing catalysts, too little condensed product was obtained for proper analysis. As shown in Figure 2, all of the other 10 wt % Co-based catalysts yielded formate, acetate, and ethanol.

The comparison of different materials used as a support for the Co-based particles shows an increase in the concentration of acetate in the presence of Ti^4+^ (1.2 mM), Al^3+^ (0.3 mM), and Mg^2+^ cations (0.2 mM) compared to pristine silica (<0.2 mM). Also, the formation of formate was favored with Co/Ti–SBA-15 (3.6 mM), and Co/Mg–SBA-15 (2.2 mM) in comparison with Co/SBA-15 (1.6 mM), while Co/Al–SBA-15 yielded a lower concentration (1.1 mM). Overall, all of the Co-based catalysts yielded metabolic intermediates from CO_2_ hydrogenation and the concentration of the central oxygenate acetate was enhanced with the modified supports compared to pristine silica. For a more comprehensive investigation of the effect of the support modification on the catalytic performance, the online-analyzed gas products are discussed in the following.

### Catalytic Performance as a Function of Co Loading and Support Material

**Figure 3:**
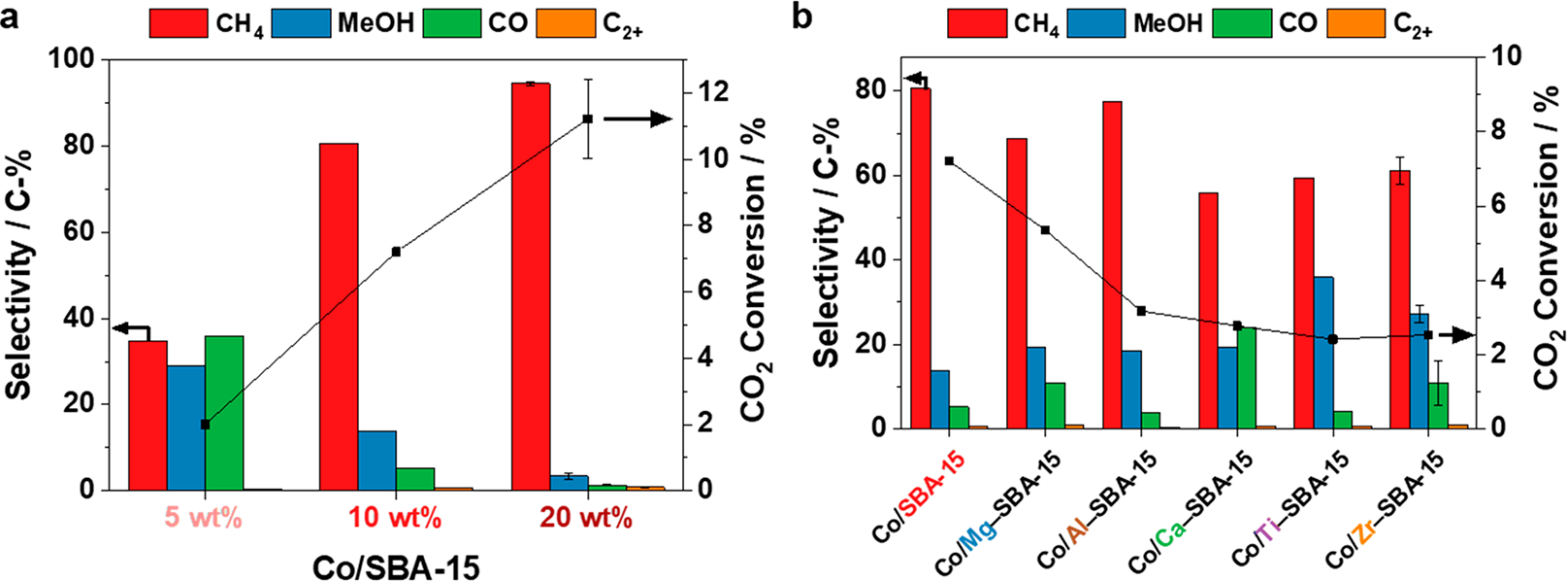
(a) Effect of Co-loading on catalytic performance of Co/SBA-15 catalysts. (b) Catalytic activity and product selectivity for CH_4_, methanol, CO, and C_2+_ hydrocarbons of 10 wt % Co/*M*–SBA-15 catalysts (*M* = Mg, Al, Ca, Ti, Zr). Reaction conditions: *T* = 180 °C, *p* = 2.0 MPa, H_2_/CO_2_/Ar = 6:3:1, 4000 cm^3^ h^–1^ g_cat_^–1^, 36 h time-on-stream. Exemplary error bars are shown based on the reproduction of the reaction with different catalyst batches. For the conversion with Co/Zr–SBA-15, the deviation is smaller than the symbol size.

The CO_2_ conversion and gas phase product selectivity were evaluated after 36 h time-on-stream as the selectivity remained stable afterward (Figure S9b). The comparison of the catalytic activity of Co/SBA-15 at different Co loadings showed an increase in the CO_2_ conversion from 2.0% to 7.2%, and 11% with increasing loading from 5 wt %, to 10 and 20 wt % Co, respectively (Figure 3a). The detected gases included CH_4_, methanol, and CO as the main products together with traces of C_2+_ hydrocarbons (<1%). This product range shows similarities to the effluents of hydrothermal vents, which also contain high concentrations of CH_4_, and other low-molecular-weight hydrocarbons.^4,7^ As seen in Figure 3a, the methane selectivity increased from 35% to 95% with increasing cobalt loading, while the selectivity toward the less hydrogenated products decreased from 29% to 3.4% in the case of methanol and 36% to 1.3% for CO. Thus, it can be inferred that the formation of the fully hydrogenated product methane was favored at lower gas flows of the reactant gas mixture relative to the amount of cobalt loading. This observation is in accordance with the decrease in the concentrations of the liquid phase products with increasing Co loading from 10 wt % to 20 wt %.

Compared to the pristine silica support, all catalysts with modified supports were less active with 5.3% CO_2_ conversion for Co/Mg–SBA-15 and similar conversions of 2.4–3.2% for the other catalysts (Figure 3b). In general, differences in the catalytic activity complicate selectivity comparison across materials, but in particular for the comparison of the Al-, Ca-, Ti-, and Zr-containing catalysts the effect of the conversion on the selectivity is considered negligible. All catalysts yielded CH_4_ as the main product with a selectivity in the range of 56–81%. The other products were again methanol, CO, and small amounts of C_2+_ hydrocarbons (0.3–1.0%). In the case of the Co/Al–SBA-15, additional traces of dimethyl ether were detected in the GC analysis. The highest selectivity toward CO was observed for the Ca-containing catalyst (24%), and the lowest, for the Ti– and Al–SBA-15-supported ones (4.0% and 3.8%, respectively). The selectivity toward methanol is of interest, as it can also serve as a methyl donor in microbial metabolism pathways.^9,17^ The Co/Ti–SBA-15 catalyst showed the highest methanol selectivity of around 36%, followed by the Zr-containing one (∼27%). The catalysts modified with Mg, Ca, and Al displayed a similar methanol selectivity of around 18–19%, and the pristine SBA-15-supported one produced only around 14% of methanol. The highest methanol selectivity of the Co/Ti–SBA-15 catalyst correlates with the highest concentrations of the more complex oxygenate products formate and acetate in the liquid phase according to HPLC analysis (Figure 2). Overall, the catalytic results revealed differences in the performance of the Co-based catalysts depending on the modification of the support material and the metal loading. However, the cause for this is yet to be elucidated by reducibility studies with H_2_-TPR, followed by DFT calculations and experimental studies on CO_2_ adsorption.

### Effect of Co–Support Interactions on Co Reducibility and Catalytic Activity

The oxidation state of supported Co-based catalysts depends strongly on the strength of Co–support interactions, and the ratio of Co and CoO phases is governed by specific Co–support linkages.^25^ While, in the case of Co supported on silica, typically a higher activity in CO_2_ hydrogenation has been reported for metallic Co compared to its oxide counterpart, the reverse trend has been reported for Co/TiO_2_.^24,27^ This was attributed to strong metal–support interactions with the partially reducible TiO_2_ support, which lead to a partial coverage of active sites by a TiO_*x*_ layer during high temperature reduction.^27^ Also the introduction of lanthanide elements into the surface of a silicate clay support has been reported to enhance the interaction with Co-based catalysts resulting in changed surface electronic properties, slightly improved reducibility, and enhanced catalytic activity in CO_2_ methanation.^52^ Considering the results of previous studies, a variation in the strength of the Co–support interactions is also expected from the modification of the silica support with the additional cations in our study, accompanied by different reducibilities of the Co-based particles.

H_2_-TPR experiments were performed to investigate the fraction of metallic Co on the support materials. Prior to the measurements, the catalysts were first calcined in an air flow at 400 °C to oxidize the Co-based particles to the spinel Co_3_O_4_ phase (see Figure S11). The TPR profiles of SBA-15 with different loadings of oxidized Co are displayed in Figure S12a. Two distinct signals between 50 and 600 °C were observed, which are characteristic of the stepwise reduction of Co_3_O_4_ to Co^0^ via the CoO intermediate.^53^ For the 5 wt % Co/SBA-15 catalyst with the smaller Co particle size, the amount of H_2_ consumed up to 600 °C was below proportion (346 μmol g^–1^) compared to the 10 and 20 wt % Co loading (1662 μmol g^–1^ and 3280 μmol g^–1^, respectively) (Table S6). Stronger Co–support interactions with the smaller sized particles could result in hardly reducible Co species. In fact, additional H_2_ consumption at temperatures above 600 °C can be associated with the reduction of strongly interacting species, such as Co–O–SiO_*x*_ or cobalt silicate.^54−56^ The relative amount of such hardly reducible cobalt species was the highest for the 5 wt % Co/SBA-15 catalyst compared to the other two metal loadings.

**Figure 4:**
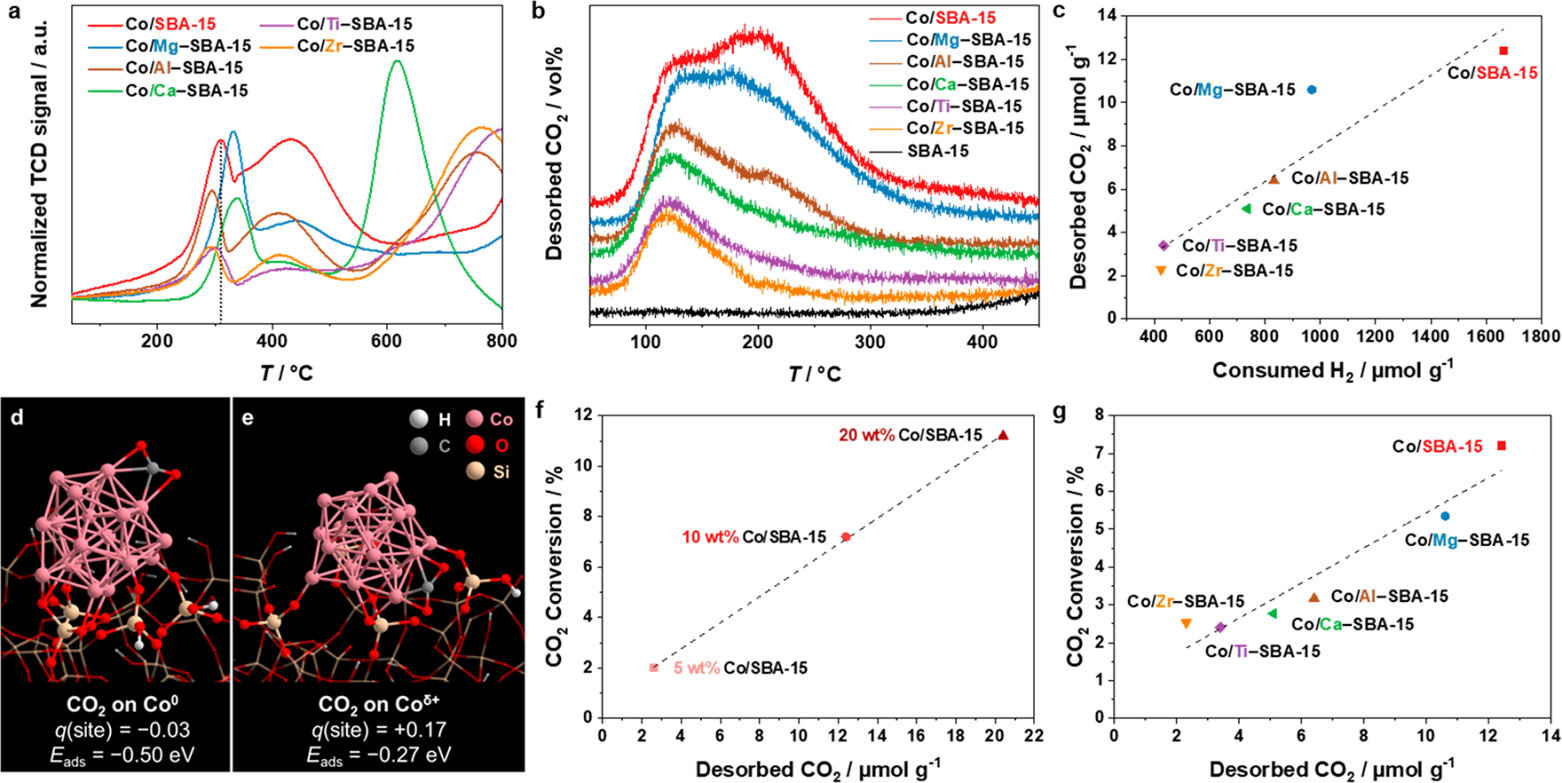
(a) H_2_-TPR profiles of Co_3_O_4_/*M*–SBA-15 catalysts and (b) CO_2_-TPD profiles of Co/*M*–SBA-15 catalysts. (c) Correlation between the amounts of consumed H_2_ in H_2_-TPR and desorbed CO_2_ in CO_2_-TPD of Co/*M*–SBA-15 materials. Adsorption structures of CO_2_ on (d) the Co^0^ and (e) the Co^δ+^ species. *q*(site) represents the sum of the Hirshfeld charges of the two cobalt atoms at the adsorption site before the interaction with CO_2_. *E*_ads_ is the adsorption energy. The Co_20_ cluster, CO_2_, and SiO_4_ units directly bonded to cobalt are highlighted as the ball-and-stick model. The other part of the silica support is shown as the wire-frame model. (f) Correlation between CO_2_ conversion and amount of desorbed CO_2_ in CO_2_-TPD of 5, 10, and 20 wt % Co/SBA-15 catalysts and (g) 10 wt % Co/*M*–SBA-15 (*M* = Mg, Al, Ca, Ti, Zr).

The TPR profiles of the Co/*M*–SBA-15 catalysts in Figure 4a in general show the same reduction pattern as the Co/SBA-15 catalysts, but the supports containing Al^3+^, Zr^4+^, and Ti^4+^ facilitated the first reduction step of Co_3_O_4_ to CoO compared to pristine Co/SBA-15 shifting the signal to lower temperatures. In contrast, in the presence of Mg and Ca sites, the reduction temperature was increased compared to the pristine silica support from 310 to 331 °C and 338 °C, respectively (see Table S7). However, the most evident difference in the TPR profiles was the more pronounced high temperature signal at *T* > 600 °C with the modified supports. Thus, the ratio of hardly reducible cobalt species strongly interacting with the support was increased by the incorporation of the additional cations.^57,58^ Although the reduction conditions of the dynamic TPR experiment differ from the static reduction process during the catalyst synthesis, the amount of H_2_ consumed up to 600 °C can serve as an indicator for the comparison of the amounts of metallic Co in the different catalysts. According to the amount of H_2_ consumed up to 600 °C, the amount of metallic cobalt on the reduced catalysts was expected to decrease in the order of SBA-15 > Mg–SBA-15 > Al–SBA-15 > Ca–SBA-15 > Ti–SBA-15 > Zr–SBA-15 (Table S7). In the literature^59^ a stronger Co–support interaction has been reported for Al_2_O_3_ compared to TiO_2_ and ZrO_2_, but the reversed order observed here might be related to the lower surface concentration of Al^3+^ compared to Ti^4+^ and Zr^4+^ as determined by XPS.

The adsorption capacity of CO_2_ on the reduced catalysts was also determined by using CO_2_-TPD. With the increase in the Co loading from 5 wt % to 20 wt %, the amount of desorbed CO_2_ increased from 2.6 μmol g^–1^ to 20.4 μmol g^–1^ CO_2_ (Figure S12b, Table S6). Thus, the CO_2_ adsorption was below proportion for the 5 wt % Co catalyst despite the smaller particle size and higher dispersion compared to the two higher loadings. Further, the 5 wt % Co catalyst displayed only weakly chemisorbed CO_2_ with the desorption centered at around 115 °C, whereas the 10 and 20 wt % catalysts contained an additional signal at 150–350 °C corresponding to moderately adsorbed CO_2_. Concerning the catalysts obtained with the modified *M*–SBA-15 supports, the Zr- and Ti-containing catalysts showed only weak CO_2_ adsorption desorbing up to around 150 °C, while the Ca- and Al-modified ones possessed additional moderately chemisorbed CO_2_ at around 180–300 °C (Figure 4b). Similar to Co/SBA-15, Co/Mg–SBA-15 adsorbed a higher amount of CO_2_ with a second desorption signal at around 160–350 °C. The individual signals in the TPD profiles can reflect different possible CO_2_ adsorption sites coexisting in the studied catalysts or different CO_2_ adsorbates. According to the H_2_-TPR results, present cobalt sites are metallic Co^0^ and hardly reducible interface-like sites, such as Co–O–SiO_*x*_ or cobalt silicate, possibly together with Co^2+^ in CoO.

To investigate the preferential adsorption sites of CO_2_, the adsorption energies of CO_2_ on metallic cobalt (Co^0^) vs oxidized cobalt (Co^δ+^) species were evaluated by DFT calculations. For simplicity, an amorphous silica surface without a pore structure was used as the local structure of the model. For the oxidized cobalt, we focused on the hardly reducible Co^δ+^–O–SiO_2_ species, which would be present under the reaction conditions after reduction at 450 °C. A Co_20_ cluster supported on amorphous silica (see SI Figure CM2) was adopted as a theoretical model of the Co/SBA-15 catalyst. Although the Co_20_ cluster is smaller than the particle size in the experiment (∼8.8 nm for the 10 wt % Co/SBA-15 catalyst), the same trend was verified for the CO_2_ adsorption with a Co_55_ cluster, which is in the range of the minimum model size for adsorption of small molecules on cobalt nanoparticles.^60^ More computational details on the model are provided in the SI in S2. Computational methods.

The Hirshfeld charge of each cobalt atom in the Co_20_/SiO_2_ model is shown in SI Figure CM3. Most of the positively charged cobalt sites are present at the interface region because of the formation of Co–O bonds with silica, while the neutral charged cobalt is located in the upper layers of the Co_20_ cluster. Thus, the Co^δ+^ species are formed due to the strong interaction with silica at the interface. Adsorption structures of CO_2_ on the top-3 positively and negatively charged adsorption sites were investigated, and the most stable adsorption structures among them are shown in Figure 4d, e. The adsorption energy of CO_2_ (*E*_ads_) on the Co^0^ site is −0.50 eV (Figure 4d), while the adsorption on the Co^δ+^ site is −0.27 eV (Figure 4e). Thus, CO_2_ binds 0.23 eV stronger on Co^0^ compared to Co^δ+^ (the definition of *E*_ads_ is given in the computational details in 2. Experimental Section). The CO_2_ adsorption on the Co–O–*M*O_*x*_ (*M* = Ti or Zr) site was also investigated (SI Figure CM4). The adsorption energies of those sites (−0.01 eV and +0.07 eV for *M* = Ti and Zr, respectively) were calculated to be weaker than that of the Co^0^ site (−0.50 eV). We note that Co^2+^ species might be also present as cobalt monoxide. However, computational investigations have shown that CO_2_ is more strongly adsorbed on metallic Co compared to CoO.^24^ Altogether, the DFT results suggest that CO_2_ preferentially adsorbs on the metallic Co sites. However, it should be kept in mind that any dynamical reconstructions of the materials under the experimental condition are not considered in our model.

For a further validation of the proposed adsorption of CO_2_ on metallic Co species, we compared the amount of desorbed CO_2_ with the H_2_ consumption during TPR (Figure 4c). The amount of H_2_ consumed up to 600 °C is used as a measure for the comparison of the amounts of metallic Co in the different catalysts. The amount of CO_2_ desorbed from the Co-based catalysts scaled with a mostly linear correlation to the H_2_ consumption (see Table S7 for the values). Thus, the chemisorption experiments agree with the DFT results and suggest metallic Co sites as the active sites for CO_2_ adsorption. Moreover, there is a strong dependence of the amount of Co^0^ on the strength of the Co–support interactions, which are modulated by the *M* sites in the Co/*M*–SBA-15 series of catalysts. A similar effect is observed with varying Co loadings due to stronger metal–support interactions with the smaller sized Co-based particles at a loading of 5 wt % Co. The CO_2_ adsorption capacity could be further correlated to the CO_2_ conversion. For the catalysts with different Co loadings and supported on modified *M*–SBA-15, the similar linear correlation between the amount of desorbed CO_2_ and its conversion was determined (Figure 4f, g). Thus, the catalytic activity is likely limited by the adsorption of CO_2_, which correlates with the amount of metallic Co and depends on the strength of the support interactions. This applied for all support materials without any exceptions.

### Support Modification Effects on Oxygenate Selectivity

The selectivity of the hydrogenation reaction of CO_2_ is governed by catalyst properties, which facilitate the generation of certain reaction intermediates or enhance their stability. The incorporation of different cations into the SBA-15 structure alters the surface properties of the materials, including the acid–base features that play a significant role in catalysis. Lewis acidic sites have been reported to guide the activity and selectivity of the most widely studied industrial copper-based CO_2_-to-methanol catalysts.^61−65^ For Cu/SiO_2_, the selectivity toward methanol formation was enhanced by the introduction of surface Lewis acid sites,^62,63^ such as Zr^4+^ and Ti^4+^, which can facilitate the formation of formate and methoxy reaction intermediates at the Cu–metal oxide interface.^64^

**Figure 5:**
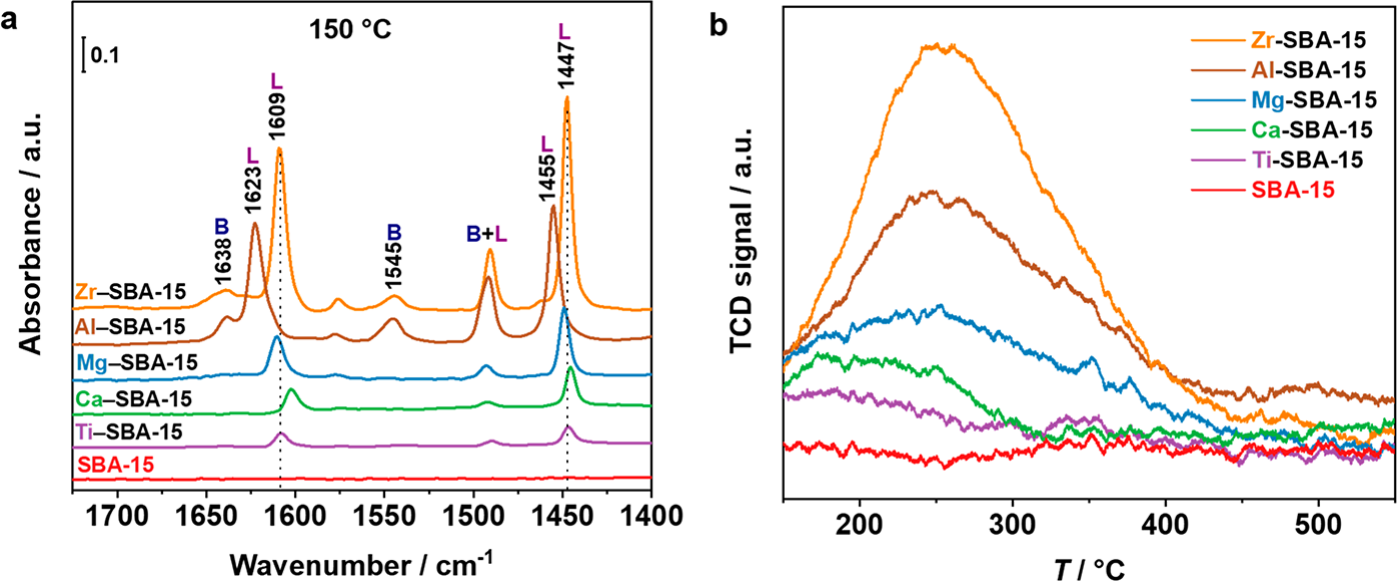
(a) FTIR spectra of adsorbed pyridine on *M*–SBA-15 support materials after evacuation at 150 °C (B: Brønsted acid sites, L: Lewis acid sites). (b) NH_3_-TPD profiles of *M*–SBA-15 support materials (*M* = Mg, Al, Ca, Ti, Zr).

Fourier-transform infrared spectroscopy was used to gain insights into the type and strength of the acid sites by using pyridine as a probe molecule, and the obtained spectra are presented in Figure 5a. The modified *M*–SBA-15 supports display characteristic bands of pyridine chemisorbed on Brønsted acid sites (BAS, 1638 and 1545 cm^–1^) and Lewis acid sites (LAS, 1600–1628 cm^–1^ and 1464–1445 cm^–1^).^66,67^ The position of the band associated with LAS evidence differences in their strength depending on the additional *M* site in the *M*–SBA-15; the higher the wavenumber, the stronger are the acid sites following the order Al–SBA-15 > Mg–SBA-15 > Zr–SBA-15 > Ti–SBA-15 > Ca–SBA-15. On the other hand, BAS are only present in the Al- and Zr-containing supports. Thus, the *M*–SBA-15 supports reveal important variations in type, and strength of the acid sites.

After Co loading, the acidity of all catalysts changed significantly and two intense and predominant bands in the LAS regions (1609 and 1450 cm^–1^) arise as seen in Figure S13. Also, the BAS almost vanished in the Co/Zr–SBA-15 and Co/Al–SBA-15 materials, suggesting possible metal–support interactions or pore blockage. A similar decrease was observed in the LAS resulting from the Al^3+^ of the support (1623 cm^–1^), being also noticeable that Co/Al–SBA-15 presents two types of acid sites with different strength (see Figure S14). The band intensity located at 1610 cm^–1^ decreased upon thermo-desorption at 250–450 °C, which indicates the moderate strength of these acid sites compared to the stronger ones, still visible at 1623 cm^–1^ and 450 °C.

For the quantification of the total amount of acid sites, NH_3_-TPD experiments were further performed. The number of acid sites decreased in the order Zr–SBA-15 > Al–SBA-15 > Mg–SBA-15 > Ca–SBA-15 > Ti–SBA-15 > SBA-15 (see Figure 5b and Table S1). The highest total amount of acid sites of Zr–SBA-15 (517 mmol NH_3_ g^–1^) can be correlated to the second highest methanol selectivity of the respective catalyst. The same way, the catalyst with the acid-free SBA-15 support showed the lowest methanol selectivity. However, the Al–SBA-15 support with the second highest number of acid sites (398 mmol NH_3_ g^–1^) and also the strongest Lewis acid sites according to the FTIR analysis only showed intermediate methanol selectivity. A too strong adsorption of the methoxy intermediate could be the reason, which would also explain the partial dehydration of methanol to dimethyl ether.^65^ However, the highest methanol selectivity of the Co/Ti–SBA-15 catalyst is opposed to its low amount of acid sites (54 mmol NH_3_ g^–1^) and the comparably weak LAS. Thus, the correlation between Lewis acidity and methanol selectivity appears less clear than for the Cu-based catalysts reported in the literature.^62−64^ Other properties of the complex catalyst system appear to also affect the selectivity by promoting the formation of certain reaction intermediates or stabilizing them.

**Figure 6:**
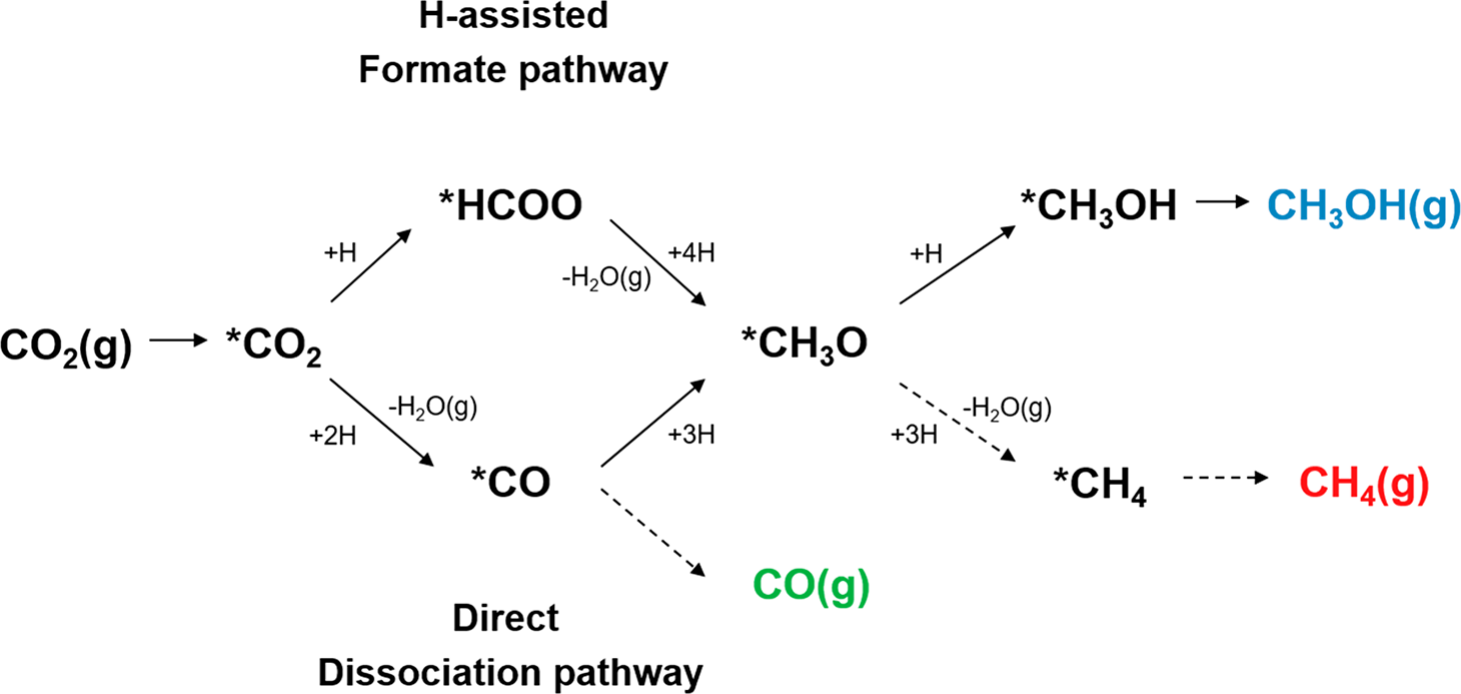
Simplified reaction pathways with key intermediates of CO_2_ hydrogenation to methanol and selected side reactions (dashed arrows). *X indicates adsorbed species.

A simplified scheme with key intermediates of the reaction pathways for the formation of methanol from CO_2_ is presented in Figure 6. The reaction can proceed either via an H-assisted pathway including a formate (*HCOO) intermediate or via a direct dissociation with the *CO intermediate.^24,68^ The preferred pathway is primarily controlled by the capability of the catalyst to bind the respective key reaction intermediates. The *CO intermediate can either desorb as CO(g) or in several consecutive reactions steps be hydrogenated to the methoxy (*CH_3_O) intermediate. This intermediate is common to both initial reaction pathways, and the final reaction step of methanol formation is either way the hydrogenation of the methoxy (*CH_3_O) species to *CH_3_OH, followed by desorption of CH_3_OH(g). A possible side reaction is the C–O bond cleavage of the *CH_3_O intermediate followed by hydrogenation to CH_4_.

The validity of the presented reaction pathways for CO_2_ hydrogenation with the Co-based catalysts in this study is supported by the reaction data at different space velocities of the reactant gas mixture presented in Figure S15. The selectivities toward CH_4_, methanol, and CO as a function of the space velocity of the reactant gas are shown exemplarily for the 10 wt % Co/SBA-15 catalyst. As both reaction products, methanol and CH_4_, are formed from the same *CH_3_O intermediate, their selectivities are interconnected. While the selectivity toward methanol is enhanced with an increasing space velocity of the CO_2_/H_2_ gas mixture, the CH_4_ selectivity decreases in an opposite manner. Thus, the hydrogenation of the *CH_3_O intermediate to methanol appears favored over the C–O bond cleavage at higher space velocities and shorter residence times of the reactant gas on the catalyst surface. As CO is formed from another reaction intermediate, the selectivity is independent of CH_4_ and methanol and constant over the variation of the reactant gas space velocity.

The importance of the generation and stabilization of the *CH_3_O intermediate in CO_2_ hydrogenation to methanol has further been stressed by a recent study on silica-supported Co-based catalysts.^25^ According to DFT calculations, the hydrogenation of the *CH_3_O intermediate to CH_4_ requires Co^0^ sites or a CoO surface with abundant oxygen vacancies.^69^ In contrast, cationic Co species bonded to the silica support at the interface have been proposed to promote the generation of the *CH_3_O intermediate and stabilize it against C–O cleavage.^25^ In this study, similarly, it could be proposed that stronger Co–support interactions with the modified silica supports result in the formation of hardly reducible cationic Co species, which might promote methanol selectivity by stabilizing the *CH_3_O intermediate against C–O cleavage and enhancing its formation. Thus, the pristine Co/SBA-15 catalyst—with the highest amount of metallic Co according to the H_2_-TPR results and consequently lowest share of cationic Co—showed the lowest methanol selectivity (see Table S7 for the values). Following the same trend, the Mg-, Al-, and Ca-modified catalysts with comparable intermediate amounts of metallic Co possess medium methanol selectivities. Finally, the methanol selectivity of the Ti- and Zr-containing materials with the lowest amounts of metallic Co showed the highest methanol selectivities. However, the difference in the methanol selectivity of the Co/Ti–SBA-15 catalyst compared to Co/Zr–SBA-15 despite the similar ratios of metallic Co^0^ and cationic Co^δ+^ indicates additional effects by other catalyst properties on the oxygenate selectivity. For instance, oxygen vacancies present in larger numbers on the surface of reducible supports, such as TiO_2_, have been reported to support the generation of the formate intermediate.^70^ Overall, the separation of the different simultaneous effects and parameters of the complex catalyst system on methanol selectivity is not very straightforward.

## 4 Conclusion

To further explore mineral catalysts in the geochemical environment of hydrothermal vents and their H_2_-dependent CO_2_ fixation, the above results have demonstrated the ability of Co-based nanoparticles supported on silica and mixed oxides to catalyze CO_2_ hydrogenation to CH_4_, methanol, and CO in the gas phase. The analysis of the condensed product phase revealed the additional formation of formate and acetate, which are key intermediates in CO_2_ fixation via the acetyl-CoA pathway. The results of this study support the function of native transition metals to catalyze metabolic reactions of early life prior to the existence of enzymes. Decreased CO_2_ hydrogenation activity was observed for the Mg-, Al-, Ca-, Ti-, and Zr-modified catalysts compared to pristine SBA-15 silica, which was correlated to the formation of strongly interacting, hardly reducible Co–O–SiO_*x*_ or cobalt silicate species over metallic cobalt. CO_2_-TPD experiments and DFT calculations for the adsorption energy of CO_2_ on a silica-supported cobalt cluster consistently suggested the preferred adsorption of CO_2_ on metallic cobalt. Despite the lower catalytic activity, the selectivity toward the metabolic oxygenates, e.g. acetate, was enhanced for the modified catalysts compared to Co/SBA-15. Overall, our findings demonstrate the versatility of native cobalt catalysts that convert CO_2_ to metabolic intermediates and underscore the natural tendency of the initial CO_2_ reducing steps of the acetyl-CoA pathway to take place in the presence of H_2_ and transition metals under laboratory simulated hydrothermal vent conditions.

## Supporting Information Available

Further experimental methods (Figure EM1–EM2), computational methods (Figure CM1–CM5), Supplementary Figures S1–S15, and Supplementary Tables S1–S7 (PDF)
