# Supplementary material for: Effects of Silica Modification (Mg, Al, Ca, Ti, and Zr) on Supported Cobalt Catalysts for H2-Dependent CO2 Reduction to Metabolic Intermediates
Source: J Am Chem Soc. 2022 Nov 9;144(46):21232–43. doi: 10.1021/jacs.2c08845 (PMC9706562; doi:10.1021/jacs.2c08845)
Supplement: Supplementary file 1 — ja2c08845_si_001.pdf [file ja2c08845_si_001.pdf]

SUPPLEMENTARY INFORMATION

Effects of Silica Modification (Mg, Al, Ca, Ti, and Zr) on Supported Cobalt Catalysts for H_2-Dependent CO_2 Reduction to Metabolic Intermediates

Kendra S. Belthle[1], Tuğçe Beyazay[1], Cristina Ochoa-Hernández[1], Ray Miyazaki[2], Lucas Foppa[2], William F. Martin[3], and Harun Tüysüz[1]*

[1]Max-Planck-Institut für Kohlenforschung, Kaiser-Wilhelm-Platz 1, 45470 Mülheim an der Ruhr, Germany

[2]The NOMAD Laboratory at the FHI of the Max-Planck-Gesellschaft and IRIS Adlershof of the Humboldt Universität zu Berlin, Faradayweg 4-6, 14195 Berlin, Germany

[3]Institute of Molecular Evolution, University of Düsseldorf, Universitätsstraße 1, 40225 Düsseldorf, Germany

*E-mail: tueysuez@kofo.mpg.de

Table of Contents

S1. Experimental Methods………………………………………………………………………… 3

Figure EM1, EM2

S2. Computational Methods……………………………………………………………………….. 5

Figure CM1–CM5

S3. Supplementary Figures……………………………………………………………………….. 9

Figure S1–S15

S4. Supplementary Tables……………………………………………………………………. 19

Table S1–S7

S5. References………………………………………………………………………………. 24

S1. Experimental Methods

Catalytic Testing for CO_2 Hydrogenation

Catalytic performance testing at 20 bar and typically 180 °C was performed in a fixed-bed reactor setup shown schematically in Figure EM1. The setup is equipped with mass-flow controllers (MFCs, Bronkhorst) to feed H_2 (Air Liquide, 99.999%), and the pre-mixed reactant gas mixture (30 vol% CO_2, 60 vol% H_2, 10 vol% Ar as internal standard, Air Liquide). The gas stream was first passed through a stainless steel (316L grade) capillary loop (length, l = 300 mm, inner diameter, i.d. = 3 mm) at 200 °C to pre-heat the reactant gas mixture. The fixed-bed stainless steel (316L grade) micro-reactor (i.d. = 12 mm) was heated by two finned copper elements (1) with two embedded 350 W heating cartridges. The temperature was controlled by two thermocouples (K-type, 0.5 mm, (2)) placed at the start and end of the catalyst bed. The catalyst bed (5) typically consisted of 850 mg of the Co-based catalyst (sieve fraction 200–400 µm grain size) diluted by 6.2 cm^3 of SiC powder (Alfa Aesar, 46 grit) for improved heat transfer. Upstream of the catalyst bed, a layer of 3.4 cm^3 of SiC powder (Alfa Aesar, 46 grit, (4)) was added for further pre-heating of the reactant gas mixture and to establish a plug-flow behavior before entering the catalyst bed. The free volume inside the reactor was reduced by two stainless steel spacers (316L grade, l = 58 mm and l = 62 mm) separated from the catalyst bed by two quartz wool plugs (3). Downstream of the reactor, the product gas stream was passed through two consecutive cold traps set to 50 °C and 100 °C at the reaction pressure to condense higher boiling oxygenate products and water. All further downstream gas lines were heated to 170 °C to prevent condensation of reaction products. When depressurized after the dome pressure regulator, the gaseous products were analyzed by an online gas chromatograph (GC, modified Agilent 7890B). The GC was equipped with two sampling loops. One loop fed into a capillary column (Restek RTX-1, 60 m) with an flame ionization detector (FID) and the other one into two consecutive packed-bed columns (HS-Q 80/120, 1 m + 3 m) equipped with a thermal conductivity detector (TCD) for the analysis of H_2, CO_2 and $C_{2\text{-}3}$ hydrocarbons. An additional TCD was used to detect Ar, CH_4 and CO, separated by a molecular sieve column (MS-5A 80/120, 3 m) along the same analysis channel. CO_2, CH_4, and CO were quantified by TCD response factors relative to Ar. CO_2 conversion (X_{CO_2}) and selectivities (S_i) were calculated from the following equations:

$$X_{CO_2}(\%) = \left(1 - \frac{A_{CO_2}/A_{Ar}}{A^0_{CO_2}/A^0_{Ar}}\right) \cdot 100\%.$$

A_{CO_2}, A_{Ar} represent the peak areas of CO_2 and Ar from the TCD during the reaction and $A^0_{CO_2}$, A^0_{Ar} the peak areas of CO_2 and Ar from the TCD during a blank measurement.

$$S_i(\%) = \left(\frac{\dot{n}_i \cdot x_i}{\sum_i \dot{n}_i \cdot x_i}\right) \cdot 100\%$$

In this equation, n_i represents the molar flow of product compound i and x_i the carbon number of the compound. The molar product flows were calculated from the TCD peak areas using the corresponding response factor[1].

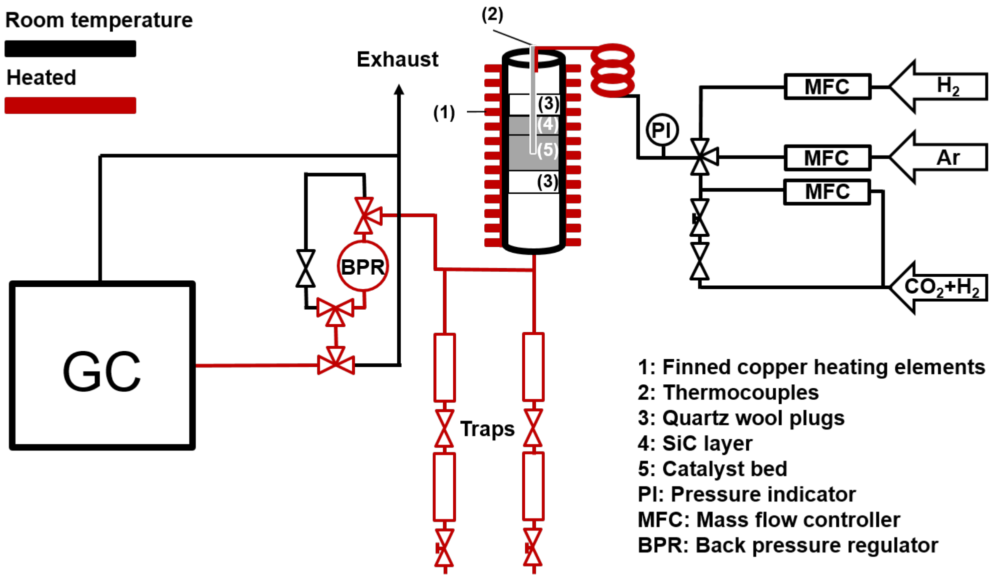

Figure EM1 Scheme flow sheet of the fixed-bed flow reactor system with online GC used to perform CO_2 hydrogenation experiments.

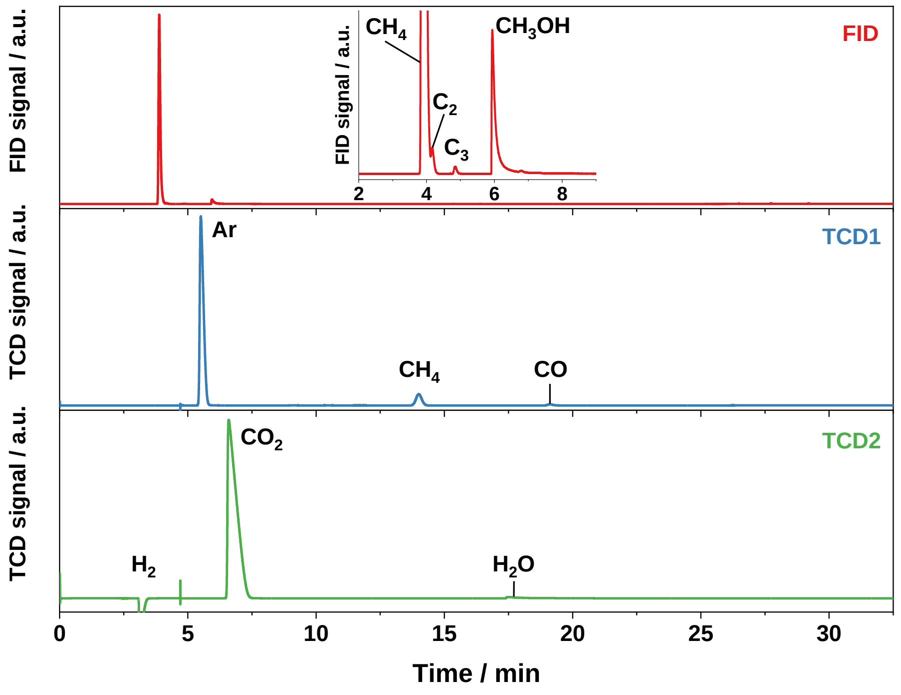

Figure EM2 Representative online gas product chromatograms for CO_2 hydrogenation with 10 wt % Co/SBA-15. Short-chain hydrocarbon products and methanol were detected with the flame-ionization detector (FID). Permanent gases such as Ar, CH_4, and CO were detected on a first thermal conductivity detector (TCD), while CO_2 and water were detected on a second TCD.

S2. Computational Methods

Details of the Co_{20}/SiO_2 Model

A Co_{20} cluster supported on the amorphous silica slab (Co_{20}/SiO_2 model) was adopted as the theoretical model of the Co/SBA-15 catalyst. The transition metal cluster was supported on the silica surface by forming –O–SiO_2 bonds with surface –OH (silanol) groups as reported in a previous theoretical study,[2] and the H_2-TPR signals at $T > 600$ °C in the present study are also indicative of such interactions. Thus, the Co_{20} was placed right above each surface –OH group with removing an H atom, and the relative stability of each structure was compared. Before the geometry optimization, the Co_{20} was rotated to maximize the number of Co–O–SiO_x bonds and H atoms were removed from the surface –OH groups within 3 Å distance from cobalt atoms. If other –OH groups interacted with cobalt (i.e., the Co–OH length became < 3 Å) during the geometry optimizations, H atoms were also removed from those –OH groups and the geometry was re-optimized. Based on this procedure, 32 structures (= the number of surface –OH groups) were generated, and the most stable one was adopted as the Co_{20}/SiO_2 model (Figure **CM*1***Figure CM2). The relative stability of all generated structures was compared by calculating the formation energy E_F according to the following equation.

$$E_F = E(\mathrm{Co_{20}/SiO_2}) + \frac{n}{2}E(\mathrm{H_2}) - E(\mathrm{Co_{20}}) - E(\mathrm{SiO_2})$$

$E(\mathrm{Co_{20}/SiO_2})$ is the potential energy of the generated Co_{20}/SiO_2 structure. $E(\mathrm{H_2})$, $E(\mathrm{Co_{20}})$ and $E(\mathrm{SiO_2})$ are the potential energies of the isolated H_2, Co_{20} cluster and silica slab, respectively. The number of the removed H atoms from the silica slab is denoted as n.

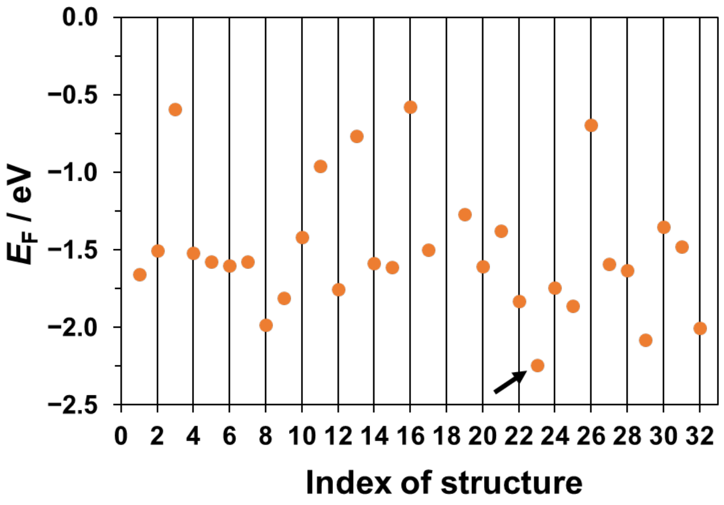

Figure CM1 Relative stability of the Co_{20} cluster on each interaction site in the silica slab. The most stable structure (No.23) is pointed out by the black arrow. Note that one surface –OH group is located below the other –OH groups, and thus, the Co_{20} cluster located at that site (No.18) could not be obtained due to the steric repulsion.

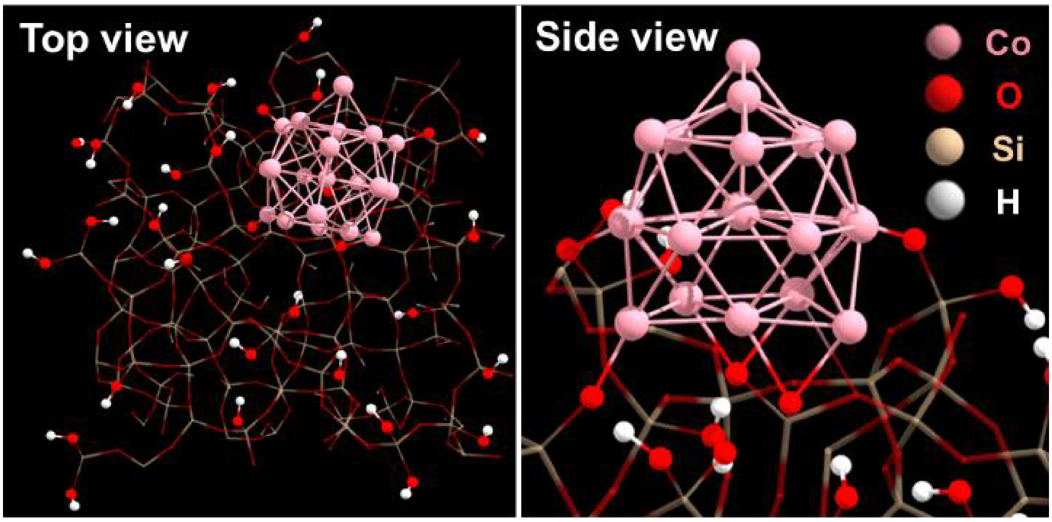

Figure CM2 Top and side view of the Co_{20}/SiO_2 model. In this figure, the Co_{20} cluster and the surface –OH groups are highlighted as the ball-and-stick model. The other part of silica is illustrated as the wire-frame model.

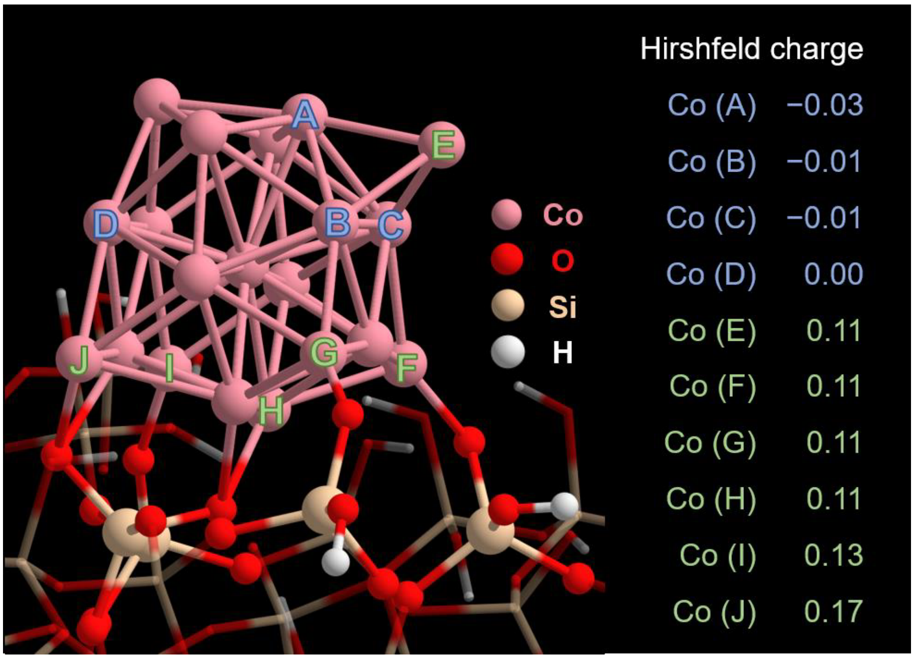

Figure CM3 Hirshfeld charge analysis for the Co_{20}/SiO_2 model. The top-3 negatively/neutral charged (A-D) and positively charged (E-J) cobalt atoms are highlighted.

CO_2 Adsorption at Co−O−MO_x (M = Ti or Zr)

To investigate the CO_2 adsorption at Co–O–MO_x (M = Ti or Zr), a Si atom in the Co_{20}/SiO_2 model was replaced by Ti or Zr because those metals tend to be in the +IV oxidation state in silica as reported in previous studies.[3, 4] As shown in Figure 4d, e and Figure CM3, four SiO_2 units are directly bonded to the Co_{20} cluster. Thus, a Si atom in one of those four SiO_2 units was replaced by a Ti or Zr atom, and the most stable structures were adopted as the theoretical models for the Co/Ti–SBA-15 and

Co/Zr–SBA-15 catalysts, respectively. All of the possible adsorption structures of CO_2 around the Co–O–MO_x site were investigated, and the most stable one is shown in Figure CM4.

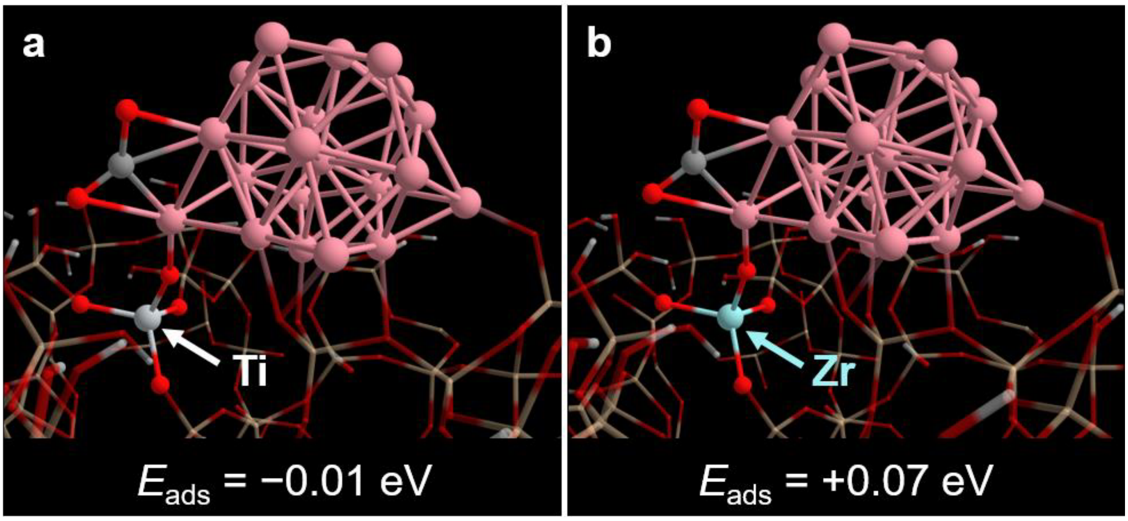

Figure CM4 Adsorption structures of CO_2 at **a)** Co–O–TiO_x and **b)** Co–O–ZrO_x. In this figure, the Co_{20} cluster, CO_2, and MO_4 (M = Ti or Zr) are highlighted as the ball-and-stick model. The other part of silica is illustrated as the wire-frame model.

Comparison between the Co_{20}/SiO_2 and Co_{55}/SiO_2 Model

The adsorption energy of CO_2 with the Co_{20} cluster was compared with a Co_{55} cluster, which corresponds to a 1 nm size cobalt particle. Although Co_{55} is also smaller than the experimental particle size (e.g. 8.8 nm for the 10 wt % Co/SBA-15 catalyst), from this range of size, the adsorption energy of small molecules (e.g. HCOOH) is almost constant against the size of the cobalt cluster.[5] The icosahedron Co_{55}, which was reported as the most stable structure in the gas phase by previous studies,[6, 7] was adopted as the initial structure of the model. The Co_{55} cluster was placed on the silica slab model at the same interaction site adopted in the Co_{20}/SiO_2 model. H atoms were removed from the surface –OH groups before and during the geometry optimizations to form the Co–O–SiO_x bonds as performed for the Co_{20}/SiO_2 model. By using the obtained Co_{55}/SiO_2 model, adsorption structures of CO_2 on the neutral and positively charged cobalt species were investigated. For the investigation of CO_2 on the neutral species, we focused on the adsorption sites around the neutral charged cobalt atom at the largest distance from the silica surface because a lot of neutral cobalt is present in the Co_{55} cluster. The most stable adsorption structure among our investigations is shown in Figure CM5. The adsorption energy on the neutral species was stronger than that of the positively charged cobalt species by 0.15 eV. Thus, both Co_{20} and Co_{55} cluster models showed the same trend for the CO_2 adsorption on the Co^0 and $Co^{\delta+}$ species although the Co_{20}/SiO_2 model tends to overestimate the adsorption energy in comparison with the Co_{55}/SiO_2 model. Note that any dynamical reconstructions of the materials under the experimental condition are not considered in our model. Additionally, we focus on the amorphous silica surface without pore structure as the local structure of SBA-15.

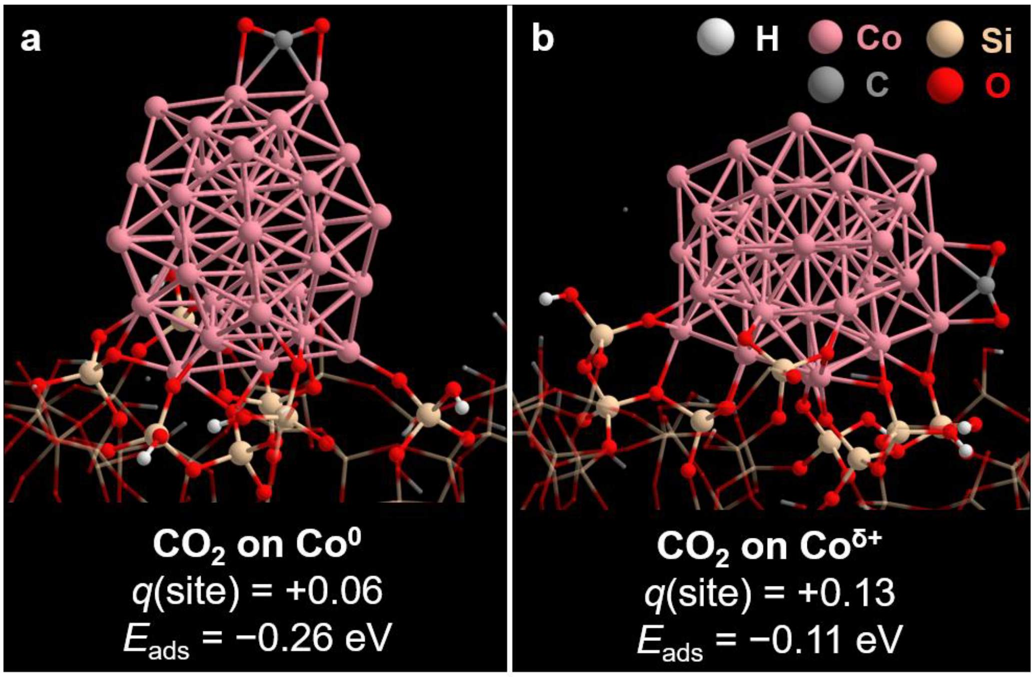

Figure CM5 Adsorption structure of CO_2 on the **a)** neutral and **b)** positively charged species of the Co_{55}/SiO_2 model. In this figure, the Co_{55} cluster, CO_2, and the SiO_4 units directly bonded to cobalt are highlighted as the ball-and-stick model. The other part of the silica support is shown as the wire-frame model.

S3. Supplementary Figures

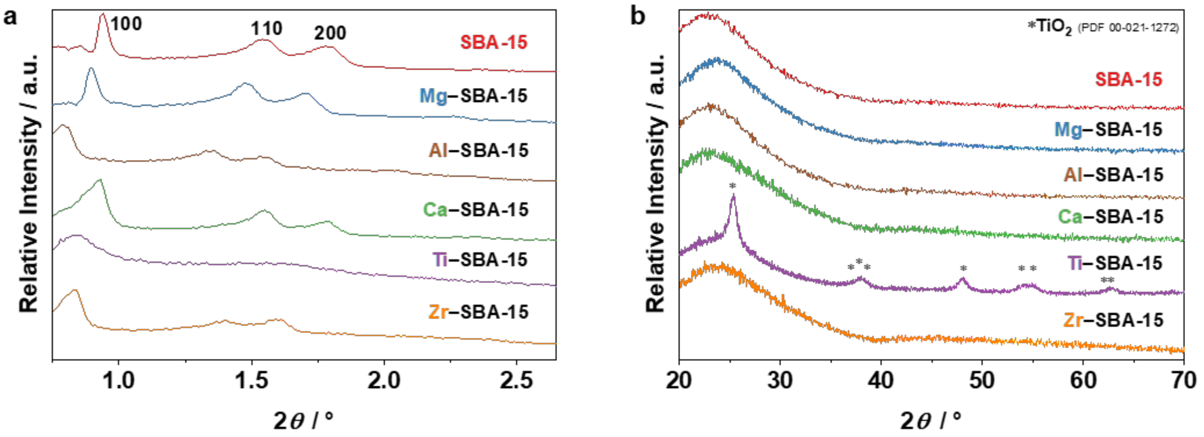

Figure S1 a) Low-angle and **b)** wide-angle XRD patterns of SBA-15 and modified *M*–SBA-15 support materials (*M* = Mg, Al, Ca, Ti, Zr).

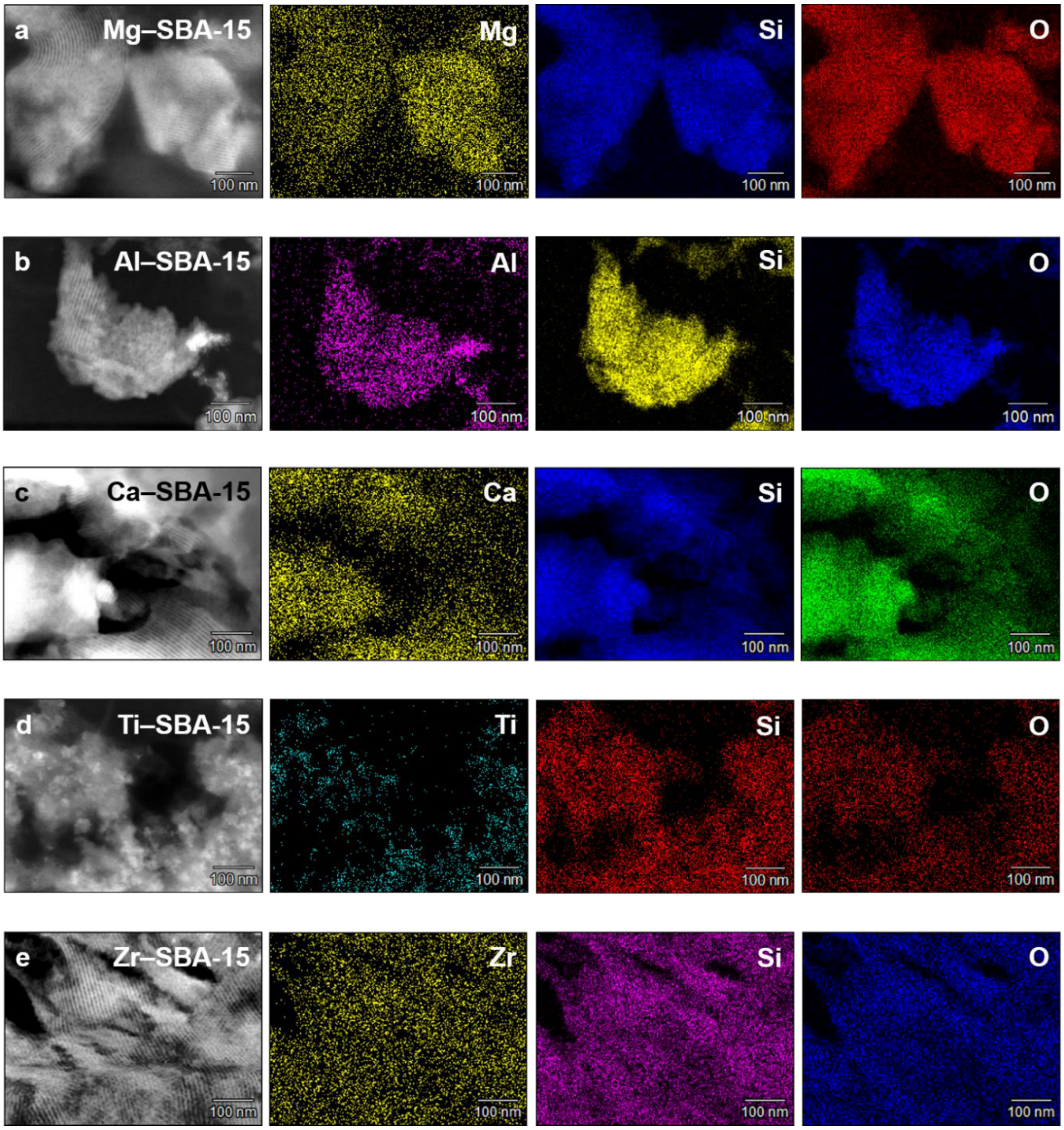

Figure S2 Dark-field HR-STEM micrographs and SEM-EDX elemental mappings of support materials **a)** Mg–SBA-15, **b)** Al–SBA-15, **c)** Ca–SBA-15, **d)** Ti–SBA-15, and **e)** Zr–SBA-15.

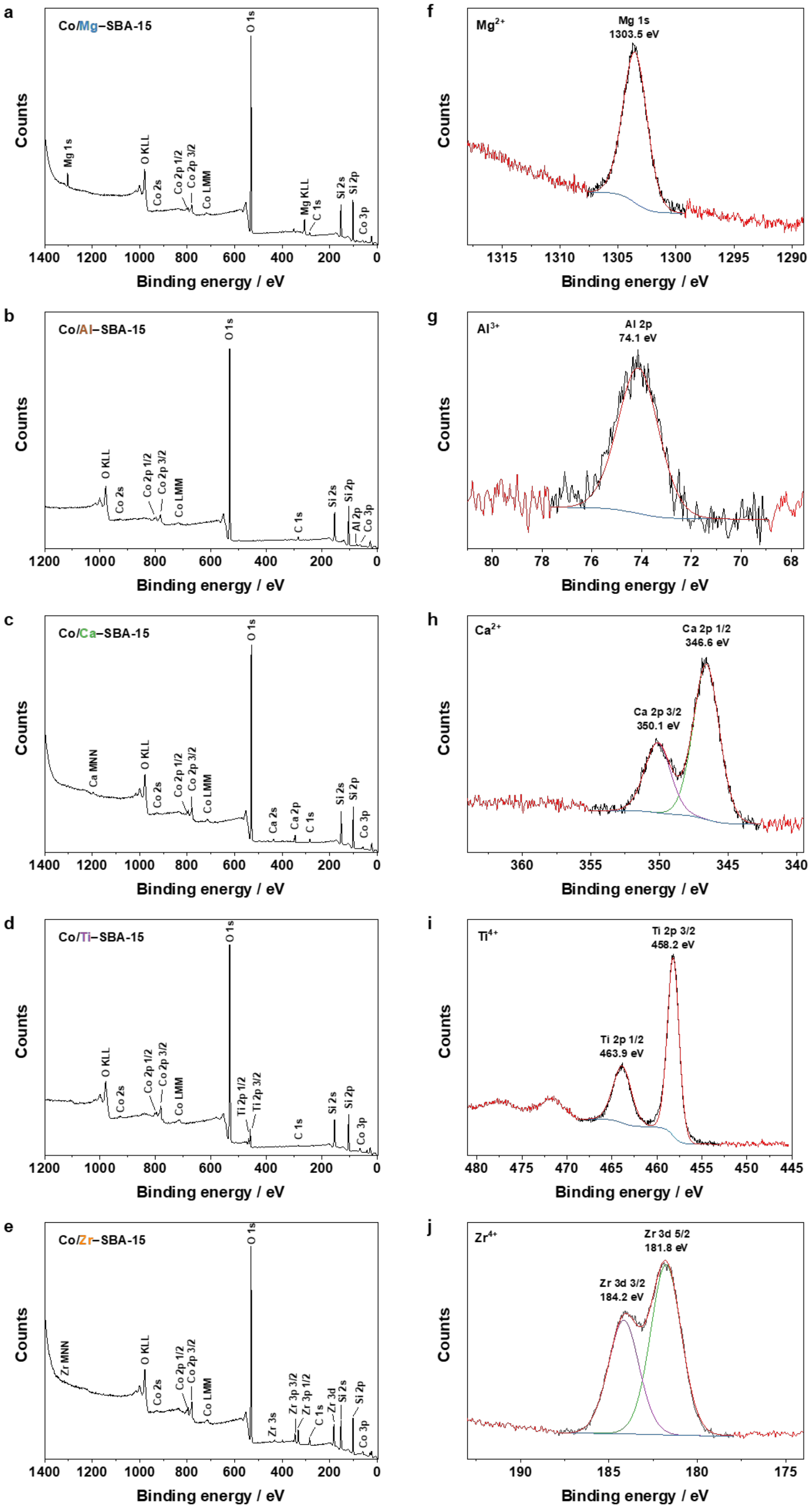

Figure S3 XPS spectra of 10 wt % **a)** Co/Mg–SBA-15, **b)** Co/Al–SBA-15, **c)** Co/Ca–SBA-15, **d)** Co/Ti–SBA-15, and **e)** Co/Zr–SBA-15 and **(f-j)** detail view of the respective hetero-atom cation regions.

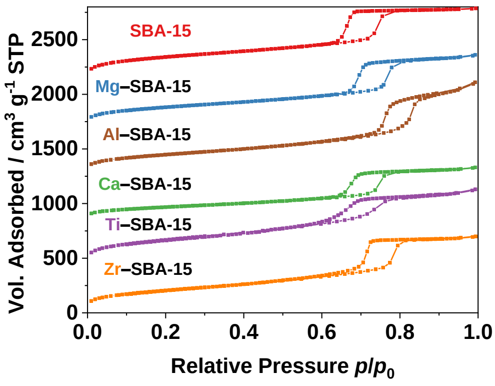

Figure S4 N_2 physisorption isotherms of SBA-15 and modified *M*–SBA-15 support materials (*M* = Mg, Al, Ca, Ti, Zr). An offset of 420 cm^3 g^{-1} was applied to the isotherms for improved clarity.

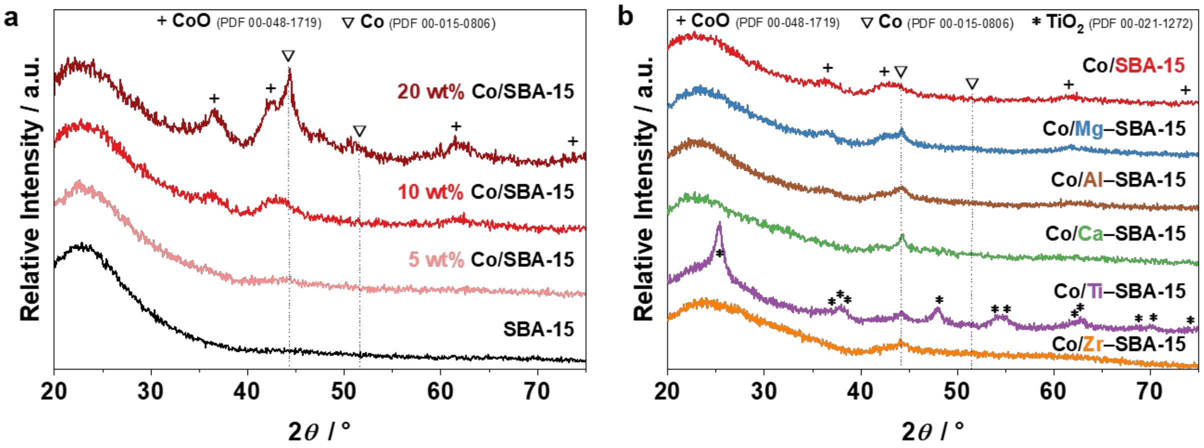

Figure S5 Wide-angle XRD patterns of **a)** 5, 10, and 20 wt % Co/SBA-15 catalysts and **b)** 10 wt % Co/*M*–SBA-15 (*M* = Mg, Al, Ca, Ti, Zr).

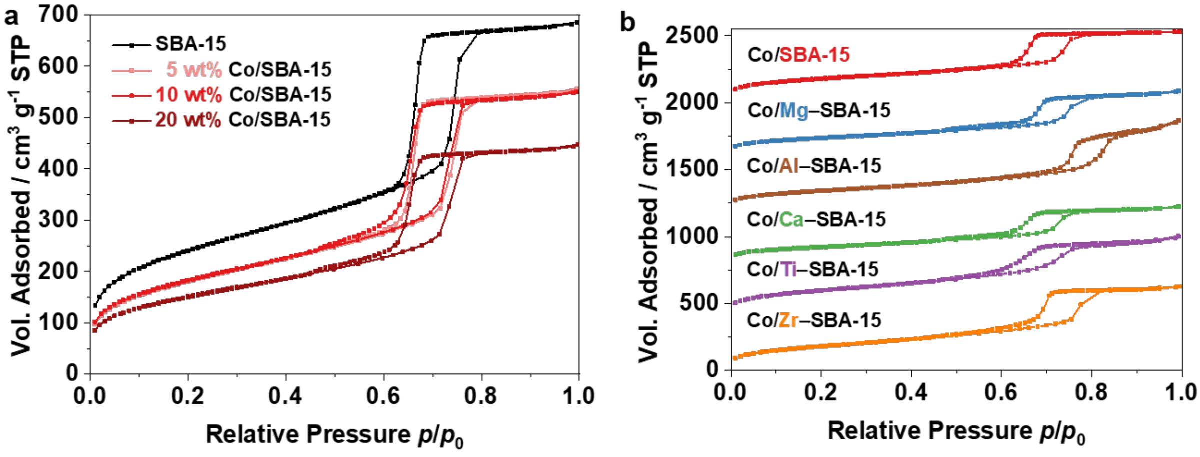

Figure S6 N_2 physisorption isotherms of **a)** 5, 10, and 20 wt % Co/SBA-15 catalysts and **b)** 10 wt % Co/*M*–SBA-15 (*M* = Mg, Al, Ca, Ti, Zr). An offset of 400 cm^3 g^{-1} was applied to the isotherms.

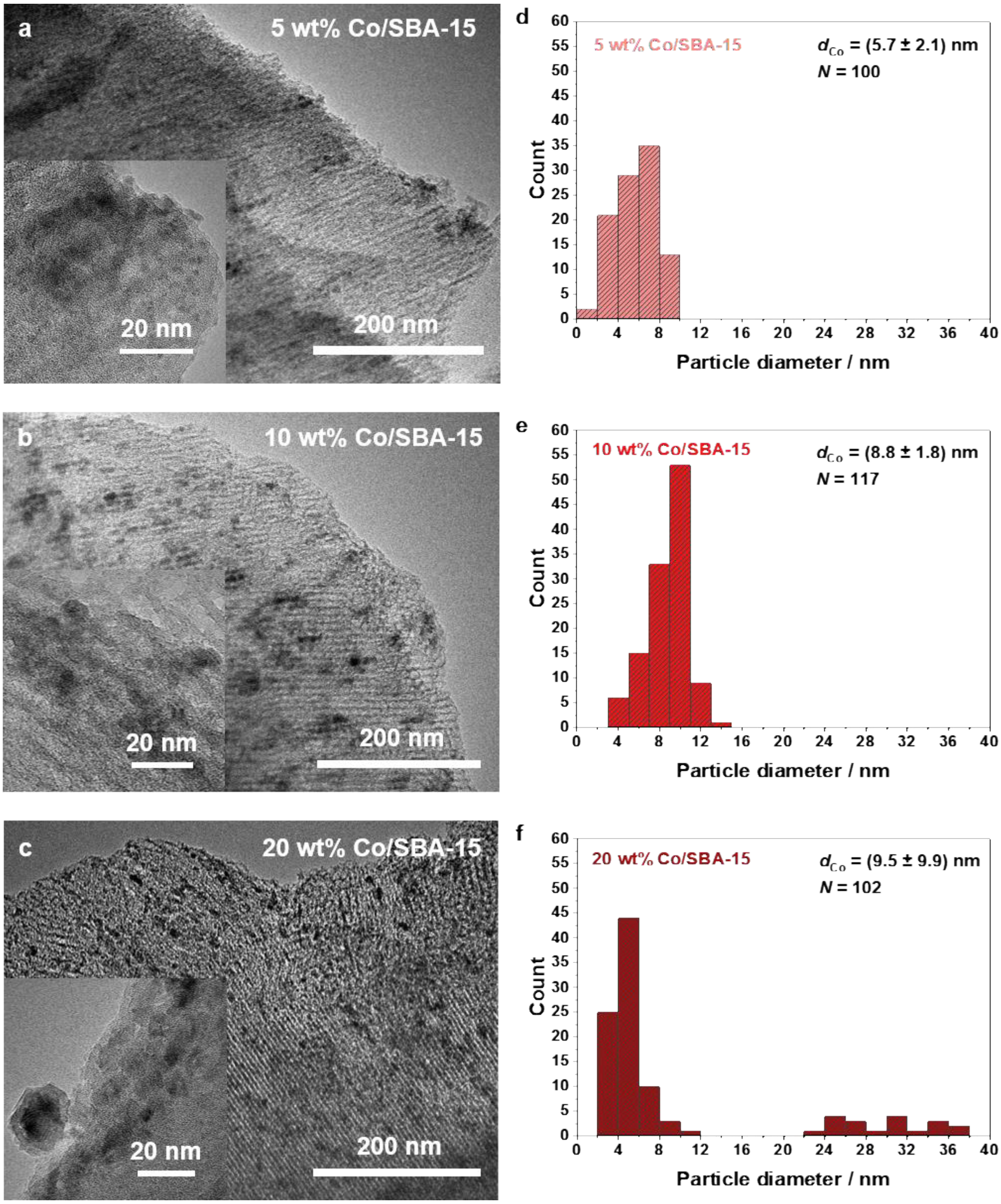

Figure S7 TEM micrographs of **a)** 5, **b)** 10, and **c)** 20 wt % Co/SBA-15 catalysts and **(d-f)** respective cobalt particle size distributions with mean cobalt particle diameter (d_{Co}) determined from the TEM micrographs and number of particles counted (N).

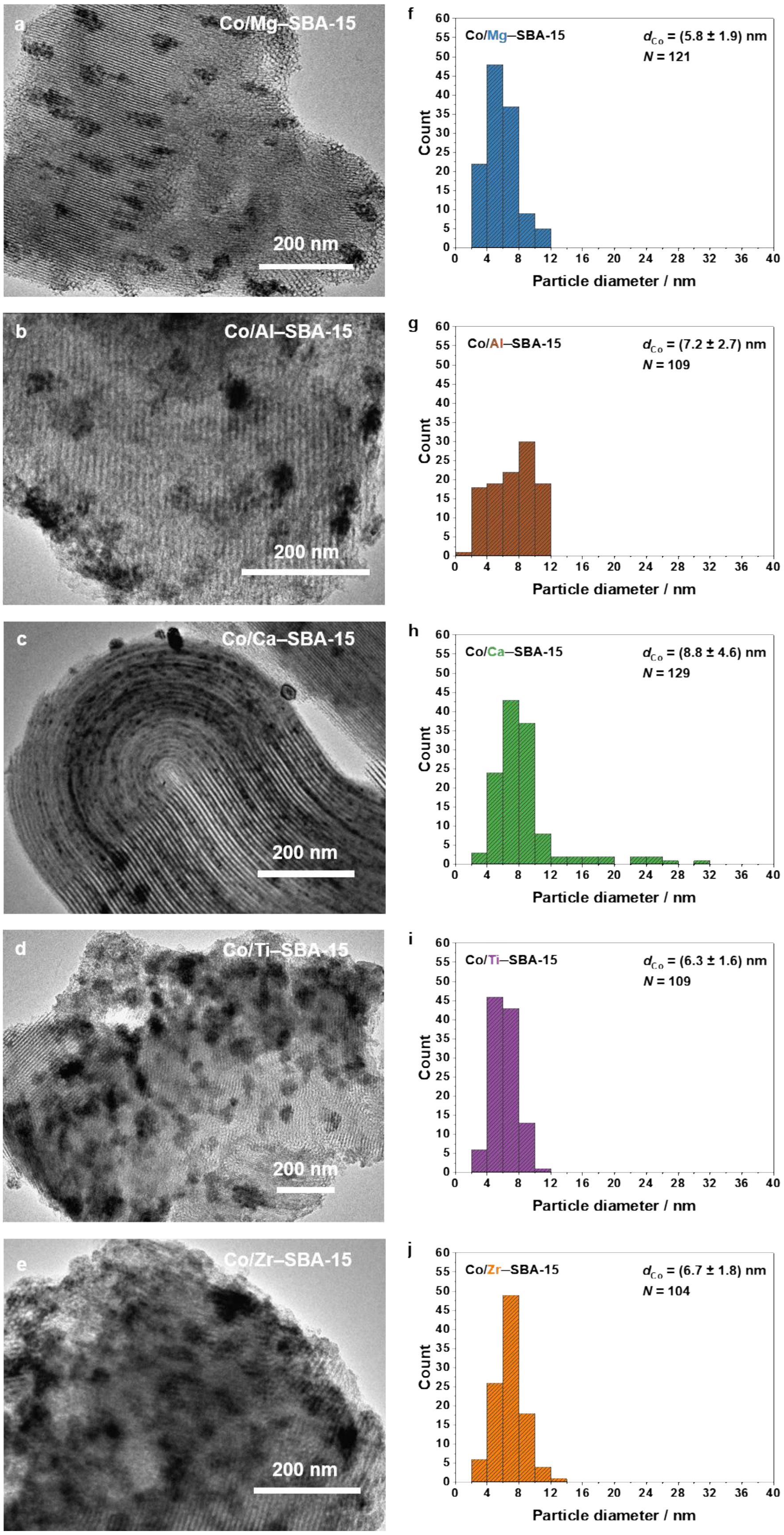

Figure S8 TEM micrographs of 10 wt % **a)** Co/Mg–SBA-15, **b)** Co/Al–SBA-15, **c)** Co/Ca–SBA-15, **d)** Co/Ti–SBA-15, and **e)** Co/Zr–SBA-15 catalysts and **(f-j)** respective cobalt particle size distributions with mean cobalt particle diameter (d_{Co}) determined from the TEM micrographs and number of particles counted (N). For some of the modified supports aggregation of the cobalt-based particles is observed, but the size of the individual particles is considered for the calculation of the particle size distribution.

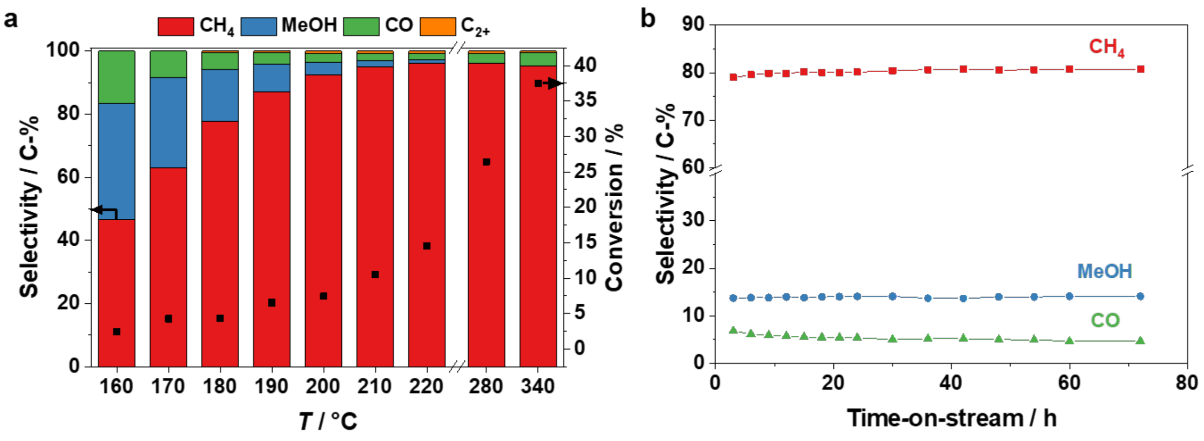

Figure S9 a) Effect of the reaction temperature on the catalytic performance of 10 wt % Co/SBA-15. Reaction conditions: p = 2.0 MPa, $H_2/CO_2/Ar$ = 6:3:1, 6000 $cm^3\ h^{-1}\ g_{cat}^{-1}$. **b)** Product selectivities as a function of time-on-stream for 10 wt % Co/SBA-15. Reaction conditions: T = 180 °C, p = 2.0 MPa, $H_2/CO_2/Ar$ = 6:3:1, 4000 $cm^3\ h^{-1}\ g_{cat}^{-1}$.

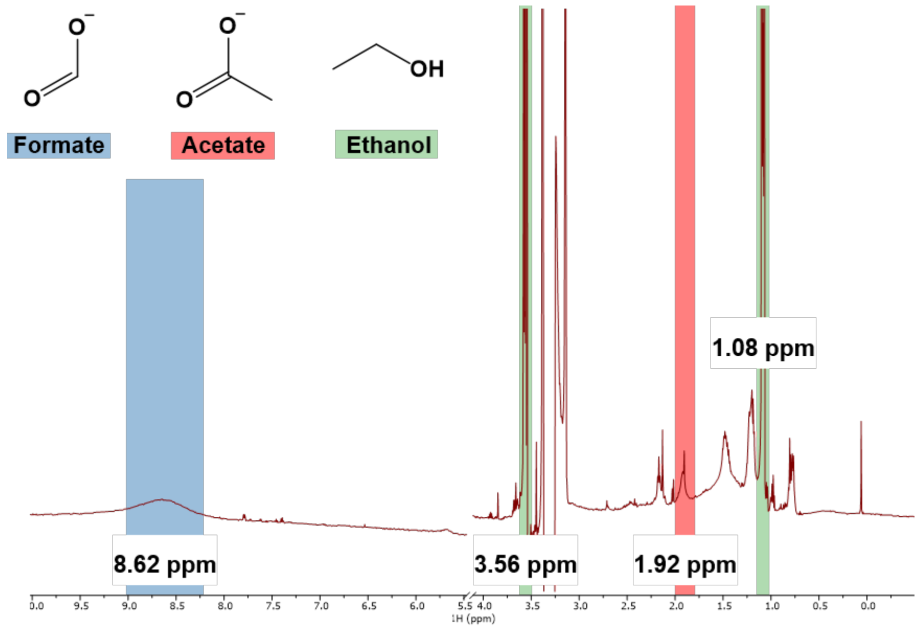

Figure S10 ^{1}H-NMR spectrum of liquid products from CO_2 hydrogenation with 10 wt % Co/SBA-15 collected after 72 h time-on-stream. Signals of water and methanol suppressed. Reaction conditions: T = 180 °C, p = 2.0 MPa, $H_2/CO_2/Ar$ = 6:3:1, 4000 $cm^3\ h^{-1}\ g_{cat}^{-1}$.

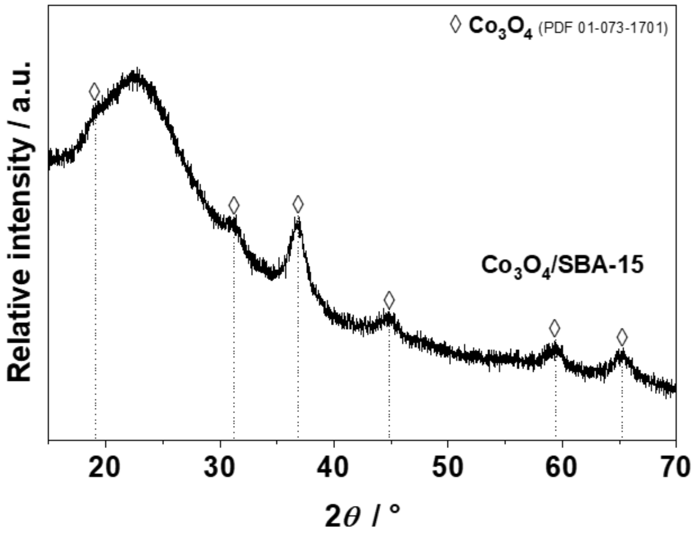

Figure S11 Wide-angle XRD pattern of the calcined 10 wt % Co/SBA-15 catalyst.

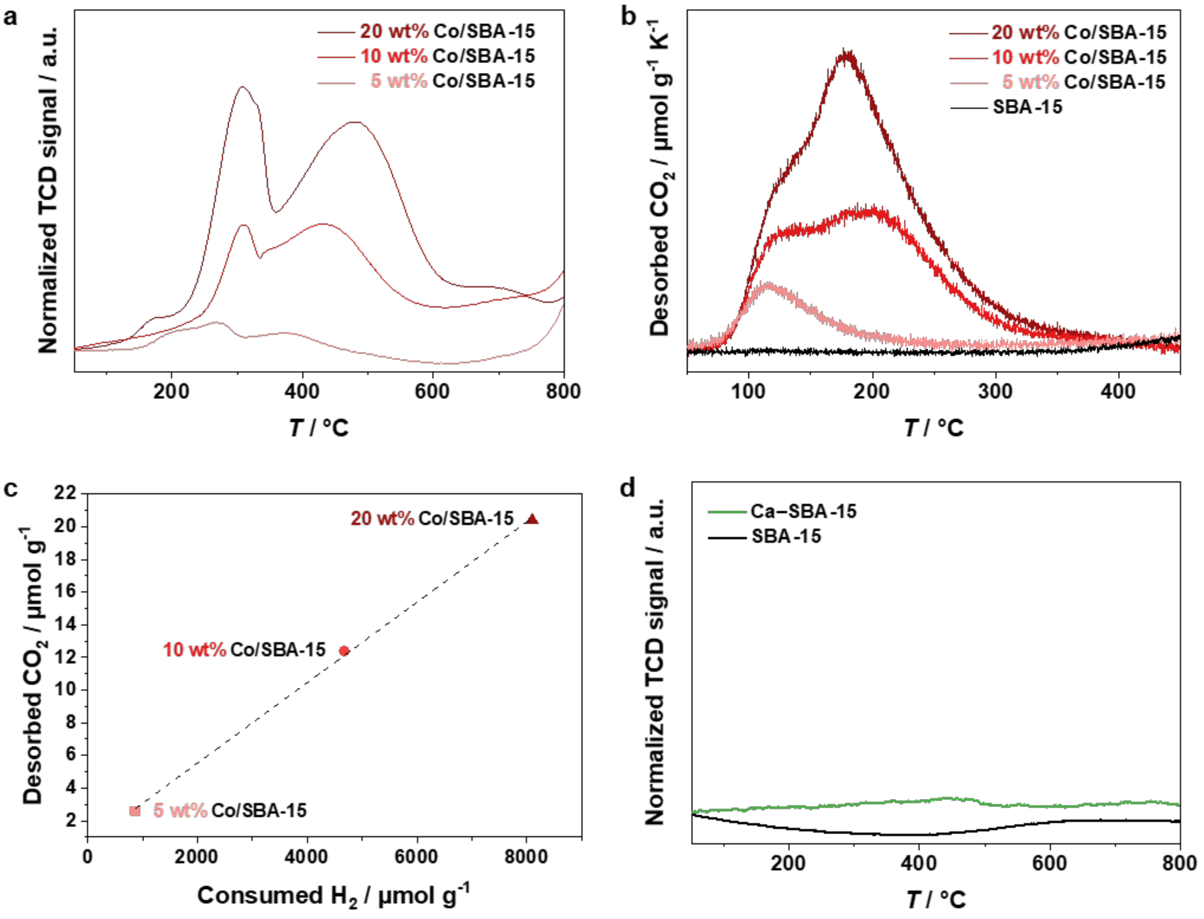

Figure S12 a) H_2-TPR profiles of Co_3O_4/SBA-15 catalysts with 5, 10, and 20 wt % Co loading. **b)** CO_2-TPD profiles of the 5, 10, and 20 wt % Co/SBA-15 catalysts. **c)** Correlation between the amounts of desorbed CO_2 in CO_2-TPD and consumed H_2 in H_2-TPR. **d)** H_2-TPR profiles of selected support materials.

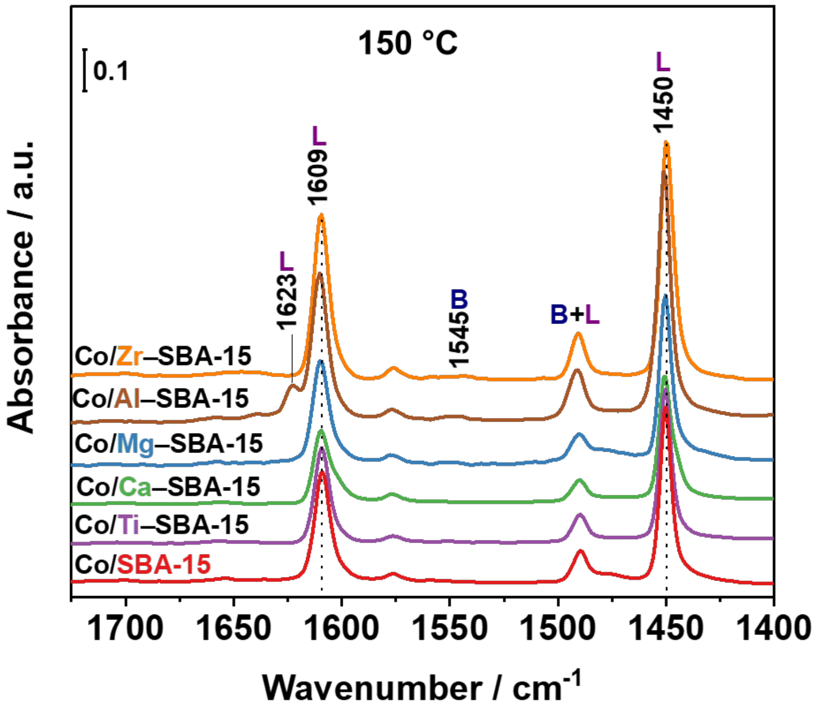

Figure S13 FT-IR spectra of adsorbed pyridine on 10 wt % Co/*M*–SBA-15 catalysts (*M* = Mg, Al, Ca, Ti, Zr) after evacuation at 150 °C (B: Brønsted acid sites, L: Lewis acid sites).

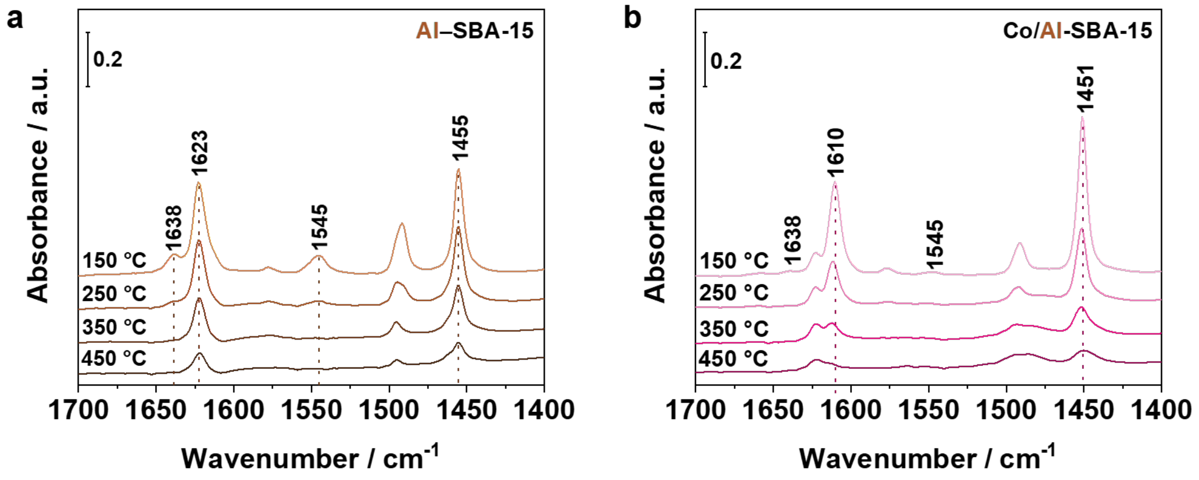

Figure S14 FT-IR spectra of adsorbed pyridine on **a)** Al–SBA-15 and **b)** 10 wt % Co/Al–SBA-15 after evacuation at 150 °C, 250 °C, 350 °C, and 450 °C.

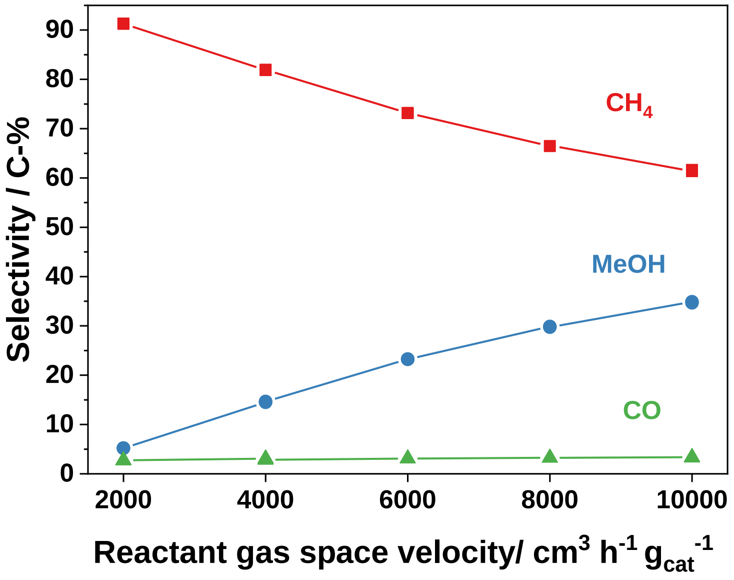

Figure S15 Selectivity towards CH_4, methanol, and CO as a function of the space velocity of the reactant gas mixture (H_2/CO_2/Ar = 6:3:1), defined as volume flow per hour and mass of catalyst, for 10 wt % Co/SBA-15 exemplarily. Reaction conditions: T = 180 °C, p = 2.0 MPa.

S4. Supplementary Tables

Table S1 Physico-chemical properties of SBA-15 and modified *M*–SBA-15 support materials (*M* = Mg, Al, Ca, Ti, Zr).

Support	Si/*M* ratio bulk[a]	Si/*M* ratio surface[b]	S_{BET}[c] ($m^2\ g^{-1}$)	V_p[d] ($cm^3\ g^{-1}$)	d_p[e] (nm)	Acidity[f] (mmol $NH_3\ g^{-1}$)
SBA-15	–	–	874	1.05	8.3	0
**Mg**–SBA-15	12	31	731	1.02	9.1	208
**Al**–SBA-15	14	31	687	1.21	12.5	398
**Ca**–SBA-15	10	22	464	0.73	8.4	110
**Ti**–SBA-15	9	11	905	1.05	7.7	54
**Zr**–SBA-15	10	10	752	1.06	9.8	517

[a] Determined from SEM–EDX.

[b] Determined from XPS.

[c] Specific surface area determined by the BET method at p/p_0 = 0.05–0.2.

[d] Total pore volume calculated from the adsorption branch at p/p_0 = 0.95.

[e] Mode of the pore diameter calculated from the adsorption branch by the BJH method.

[f] Determined from NH_3-TPD.

Table S2 Physico-chemical properties of the 5, 10, and 20 wt % Co/SBA-15 catalysts.

Catalyst	Co loading[a] (wt %)	S_{BET}[b] ($m^2\ g^{-1}$)	V_p[c] ($cm^3\ g^{-1}$)	d_p[d] (nm)
**20 wt %** Co/SBA-15	24.0	547	0.68	7.7
**10 wt %** Co/SBA-15	12.7	665	0.84	7.7
**5 wt %** Co/SBA-15	6.0	659	0.85	8.4
SBA-15	–	874	1.05	8.3

[a] Determined from bulk SEM–EDX.

[b] Specific surface area determined by the BET method at p/p_0 = 0.05–0.2.

[c] Total pore volume calculated from the adsorption branch at p/p_0 = 0.95.

[d] Mode of the pore diameter calculated from adsorption branch by the BJH method.

Table S3 Physico-chemical properties of the 10 wt % Co/*M*–SBA-15 catalysts (*M* = Mg, Al, Ca, Ti, Zr).

**Catalyst**	**Co loading[a] (wt %)**	**S_{BET}[b] (m^2 g^{-1})**	**V_p[c] (cm^3 g^{-1})**	**d_p[d] (nm)**
Co/SBA-15	12.7	665	0.84	7.7
Co/**Mg**–SBA-15	11.0	501	0.73	8.4
Co/**Al**–SBA-15	11.7	518	0.95	11.9
Co/**Ca**–SBA-15	11.0	318	0.56	8.3
Co/**Ti**–SBA-15	11.1	724	0.88	7.7
Co/**Zr**–SBA-15	11.6	664	0.95	9.1

[a] Determined from bulk SEM–EDX.

[b] Specific surface area determined by the BET method at p/p_0 = 0.05–0.2.

[c] Total pore volume calculated from the adsorption branch at p/p_0 = 0.95.

[d] Mode of the pore diameter calculated from the adsorption branch by the BJH method.

Table S4 Concentrations of oxygenate products determined by HPLC from CO_2 hydrogenation with 10 wt % Co/SBA-15 at various temperatures collected after 24 h at the respective temperature. Reaction conditions: p = 2.0 MPa, $H_2/CO_2/Ar$ = 6:3:1, 6000 $cm^3\ h^{-1}\ g_{cat}^{-1}$.

T (°C)	*c*(formate) (mM)	*c*(acetate) (mM)	*c*(methanol) (mM)	*c*(ethanol) (mM)
180	1.3	detected	463	1.1
190	1.3	not detected	436	2.0
200	0.9	not detected	301	2.6
210	0.4	not detected	193	3.0
220	0.2	detected	118	2.8
280	0.2	detected	15	0.2
340	detected	not detected	1	not detected

Table S5 Concentrations of oxygenate products determined by HPLC from CO_2 hydrogenation with 10 and 20 wt % Co/SBA-15 collected after 72 h time-on-stream. Reaction conditions: T = 180 °C, p = 2.0 MPa, $H_2/CO_2/Ar$ = 6:3:1, 4000 $cm^3\ h^{-1}\ g_{cat}^{-1}$.

Co loading (wt %)	*c*(formate) (mM)	*c*(acetate) (mM)	*c*(ethanol) (mM)
10	1.6	detected	2.0
20	0.4	detected	1.5

Table S6 Amount of consumed H_2 from H_2-TPR of Co_3O_4/SBA-15 catalysts (up to T = 600 °C) and desorbed CO_2 from CO_2-TPD (up to T = 450 °C) of the 5, 10, and 20 wt % Co/SBA-15 catalysts.

**Catalyst**	**Consumed H_2 (μmol g^{-1})**	**Desorbed CO_2 (μmol g^{-1})**
**5 wt %** Co/SBA-15	346	2.6
**10 wt %** Co/SBA-15	1662	12.4
**20 wt %** Co/SBA-15	3280	20.4

Table S7 Temperature of first reduction signal and amount of consumed H_2 from H_2-TPR of the Co_3O_4/*M*–SBA-15 catalysts (up to T = 600 °C) and desorbed CO_2 from CO_2-TPD (up to T = 450 °C) of the 10 wt % Co/*M*–SBA-15 catalysts (*M* = Mg, Al, Ca, Ti, Zr).

**Catalyst**	**T_{red} (°C)**	**Consumed H_2 (μmol g^{-1})**	**Desorbed CO_2 (μmol g^{-1})**
Co/SBA-15	310	1662	12.4
Co/**Mg**–SBA-15	331	971	10.6
Co/**Al**–SBA-15	295	833	6.4
Co/**Ca**–SBA-15	338	736	5.1
Co/**Ti**–SBA-15	300	434	3.4
Co/**Zr**–SBA-15	295	425	2.3

S5. References

1. Dietz, W. A., Response factors for gas chromatographic analyses. *Journal of Chromatographic Science* **1967,** *5* (2), 68-71.
2. Ewing, C. S.; Hartmann, M. J.; Martin, K. R.; Musto, A. M.; Padinjarekutt, S. J.; Weiss, E. M.; Veser, G.; McCarthy, J. J.; Johnson, J. K.; Lambrecht, D. S., Structural and Electronic Properties of Pt13 Nanoclusters on Amorphous Silica Supports. *The Journal of Physical Chemistry C* **2015,** *119* (5), 2503-2512.
3. Perez-Beltran, S.; Balbuena, P. B.; Ramírez-Caballero, G. E., Surface Structure and Acidity Properties of Mesoporous Silica SBA-15 Modified with Aluminum and Titanium: First-Principles Calculations. *The Journal of Physical Chemistry C* **2016,** *120* (32), 18105-18114.
4. Dong, X.; Lu, J.; Yu, Y.; Zhang, M., A DFT study on Zr-SBA-15 catalyzed conversion of ethanol to 1,3-butadiene. *Physical Chemistry Chemical Physics* **2018,** *20* (18), 12970-12978.
5. Farkaš, B.; Terranova, U.; de Leeuw, N. H., Binding modes of carboxylic acids on cobalt nanoparticles. *Physical Chemistry Chemical Physics* **2020,** *22* (3), 985-996.
6. Farkaš, B.; de Leeuw, N. H., Towards a morphology of cobalt nanoparticles: size and strain effects. *Nanotechnology* **2020,** *31* (19), 195711.
7. Rodríguez-López, J. L.; Aguilera-Granja, F.; Michaelian, K.; Vega, A., Structure and magnetism of cobalt clusters. *Physical Review B* **2003,** *67* (17), 174413.
